# Simultaneous spatio-temporal matching pursuit decomposition of evoked brain responses in MEG

**DOI:** 10.1007/s00422-016-0707-5

**Published:** 2017-01-21

**Authors:** Paweł Kordowski, Artur Matysiak, Reinhard König, Cezary Sielużycki

**Affiliations:** 1 Biomedical Physics Division, Institute of Experimental Physics, Faculty of Physics, University of Warsaw, ul. Pasteura 5, 02-093 Warsaw, Poland; 2 Special Lab Non-Invasive Brain Imaging, Leibniz Institute for Neurobiology, Brenneckestr. 6, 39118 Magdeburg, Germany; 3 Control of Normal and Abnormal Movements Team, ICM Brain and Spine Institute, Pierre-and-Marie-Curie University (Paris VI, Sorbonne), INSERM UMR1127, CNRS UMR7225, Hôpital Pitié Salpêtrière, 47 bd de l’Hôpital, 75013 Paris, France; 4 Faculty of Computer Science and Management, Wrocław University of Science and Technology, Wybrzeże Wyspiańskiego 27, 50-370 Wrocław, Poland; 5 Department of Biomedical Engineering, Faculty of Fundamental Problems of Technology, Wrocław University of Science and Technology, Wybrzeże Wyspiańskiego 27, 50-370 Wrocław, Poland

**Keywords:** Evoked responses, M100, Magnetoencephalography (MEG), Matching pursuit, Spatio-temporal decomposition

## Abstract

We present a novel approach to the spatio-temporal decomposition of evoked brain responses in magnetoencephalography (MEG) aiming at a sparse representation of the underlying brain activity in terms of spatio-temporal atoms. Our approach is characterized by three attributes which constitute significant improvements with respect to existing approaches: (1) the spatial and temporal decomposition is addressed simultaneously rather than sequentially, with the benefit that source loci and corresponding waveforms can be unequivocally allocated to each other, and, hence, allow a plausible physiological interpretation of the parametrized data; (2) it is free from severe a priori assumptions about the solution space; (3) it comprises an optimization technique for the use of very large spatial and temporal subdirectories to greatly reduce the otherwise enormous computational cost by making use of the Cauchy–Schwarz inequality. We demonstrate the efficiency of the approach with simulations and real MEG data obtained from a subject exposed to a simple auditory stimulus.

**Electronic supplementary material:**

The online version of this article (doi:10.1007/s00422-016-0707-5) contains supplementary material, which is available to authorized users.

## Sparse representation of evoked brain responses by means of adaptive approximation procedures

In magnetoencephalography (MEG, Hämäläinen et al. 1993) and electroencephalography (EEG, Schomer and Lopes da Silva 2010), evoked magnetic fields or electric potentials of neural activity are measured extracranially with excellent temporal resolution. Accurate and unique localization of the underlying source(s), however, suffers from the ill-posed inverse problem inherent to the two techniques. Over the years, numerous different source localization approaches have been introduced, which included the equivalent current dipole (ECD) model, distributed source model, beamformers, etc. (see Darvas et al. 2004; Wendel et al. 2009, and references therein). What all these methods have in common is that they have to deal with the problem of unambiguity, reproducibility, and computational efficiency; these features determine to a large extent the success of a method.

Recently, new approaches which make use of sparse representations of MEG/EEG signals have increasingly become the focus of attention, for example in several variations of the matching pursuit (MP) algorithm originally published by Mallat and Zhang (1993) in their seminal work. MP is an adaptive approximation procedure applied to the data of interest, for example time series in MEG or EEG recordings. MP iteratively decomposes these data such that a linear expansion of so-called atoms—each of which represents a distinct component of the underlying data—and an unexplained residual assumed to represent noise are obtained. The atoms are elements of a large set of functions commonly referred to as dictionary. Most often Gabor functions are used, since they offer the best trade-off between time and frequency resolution. In the work of Geva (1996), as well as of Durka et al. (2005) and Matysiak et al. (2005), temporal and spatial aspects of EEG signal parametrization and source localization were handled sequentially. In these approaches, trial-averaged waveforms of multichannel EEG signals were first decomposed using multichannel MP (MMP) with large time–frequency dictionaries before, in a second step, a search for the optimal location of the sources was performed. These approaches differ from each other inasmuch as Geva (1996) used a dictionary of Hermite functions and a discrete source model (ECDs), whereas Durka et al. (2005) and Matysiak et al. (2005) used a large dictionary of Gabor functions as preprocessing step to distributed source analysis.

Gratkowski et al. (2008) parametrized multichannel waveforms by means of a space–time–frequency MP algorithm. The atoms used in this approach had additional spatial properties; they were constructed from spatial modes computed by means of Bessel functions modulated in time with Gabor functions. In two consecutive publications of this group (Lelic et al. 2009, 2011), multichannel MP was successfully used as preprocessing step for inverse solutions with ECDs. Gramfort et al. (2013b) addressed the inverse problem in MEG/EEG source localization in an approach which was also based on the time–frequency characteristics of the elements of Gabor dictionaries. They proposed to make use of mixed norms defined in terms of time–frequency decompositions of MEG/EEG sources to localize focal non-stationary sources and demonstrated the efficiency of their approach in which spatial sparsity, temporal smoothness, and non-stationarity of the source signals could be recovered.

Wu and Swindlehurst (2013) claimed in their work that the use of multichannel MP with spatial dictionaries is an approach superior to ECD localization; however, they did not consider temporal aspects in the signal decomposition. Other widely accepted methods which are not based on an MP decomposition of the measured data, like FOCUSS (Gorodnitsky et al. 1995) or RAP-MUSIC (Mosher and Leahy 1999), also address the location of the underlying ECDs and the derivation of the corresponding waveforms in separate steps. This is done either for all sources simultaneously, or one by one.

Here, we introduce a novel, hybrid MP algorithm to decompose the time course of evoked magnetic fields into distinct components and localize the underlying brain activity of each of these components without heavy a priori assumptions about the solution space. We termed this approach spatio-temporal matching pursuit (STMP), since—contrary to the aforementioned approaches in which spatial and temporal decompositions of the MEG signal are performed *sequentially*—STMP *simultaneously* realizes a spatial and temporal decomposition of the MEG signal in each iteration by means of a spatio-temporal dictionary. STMP is a source localization strategy for MEG/EEG data in which each signal component represents the activity of exactly one dipolar source.

The STMP dictionary is composed of combinations of the elements of a large set of dipolar fields (spatial subdictionary), each originating from an ECD located on the surface of the tessellated cortex, to address the spatial aspect of the MEG data to be parametrized, and of the elements of a large set of chirplets (temporal subdictionary) to address the temporal aspect. For each iteration of the STMP algorithm, the match between the current data residuum and the elements of the spatio-temporal dictionary is maximized. Ultimately, one obtains a sparse representation of the analysed multivariate signal as a linear expansion of spatio-temporal atoms, each of which represents an ECD with an individual time course. Furthermore, our implementation of the proposed STMP approach also allows the use of spatial and temporal subdictionaries with very large numbers of elements, way more than 100,000. In order to vastly reduce the associated immense computational effort, we introduce a very efficient strategy based on the use of the Cauchy–Schwarz inequality. As a consequence, results with high spatial and temporal resolution are obtained in a few minutes on a modern powerful computer.

We evaluated the feasibility of this novel approach by using simulated data, and by applying it to an illustrative MEG data set obtained from a measurement in which the subject was exposed to a repeated auditory stimulation with sinusoidal 1-kHz tones. We focused on a time window around the most prominent late auditory evoked magnetic field (AEF), the M100, with a peak latency of approximately 100 ms after stimulus onset. Furthermore, we assessed the robustness of the proposed method by studying its performance on several smaller subsets of trials, for different signal-to-noise ratios (SNRs), and for up to 10 ECDs. In addition, we compared the performance of STMP with that of a well-established source localization method, the RAP-MUSIC algorithm (Mosher and Leahy 1999).

## Simultaneous decomposition of MEG/EEG signals in space and time

The basic idea behind the adaptive approximation by means of matching pursuit is that the signal to be analysed—which, in the context of this work, is a trial-averaged MEG or EEG signal—is explained by (i.e. decomposed into) a relatively small subset of functions (atoms) chosen from a redundant dictionary to optimally fit to the local signal structures. Here, optimality is understood as minimizing the error of the representation with respect to the signal to be analysed. Since the optimal choice for a given number of atoms is an NP-hard problem (Davis 1994), MP only provides a suboptimal solution as the result of an iterative decomposition procedure. In the first iteration, the atom that best matches the analysed signal is chosen from the dictionary. In each consecutive iteration, the atom that best matches the residual, i.e. the signal obtained after subtracting the results of the previous iterations, is chosen. For a complete dictionary, such an iterative procedure converges to the original signal, but finite expansions are used in practice. Various strategies exist to determine a plausible threshold as to the number of atoms in the final representation; here, we used a simple energetic criterion (see Sect. 3.2.1).

### Spatio-temporal dictionary

The spatio-temporal dictionary is composed of two separate sets. The spatial subdictionary *D* contains real-valued dipolar field distributions generated by computing the forward solutions of a large number of ECDs onto a 3-D mesh of sensors of an MEG system which, in our case, was the 4D-Neuroimaging Magnes 2500 whole-head device with 148 magnetometer sensors. Hence, the spatial subdictionary is an $N_d \times N_c$ matrix of $N_d$ *spatial atoms* and $N_c$ MEG channels. Each spatial atom is normalized such that its $\ell ^2$ norm is equal to 1. The forward solutions were computed using a realistic head model with the spherical-harmonics approximation (Nolte 2003) of the subject’s magnetic-resonance-imaging (MRI) scan, as implemented in the FieldTrip toolbox (Oostenveld et al. 2011). Distinct dipole locations were obtained by a triangulation of the cortex using the hybrid watershed and deformable surface algorithm (Ségonne et al. 2004) of the FreeSurfer software (http://surfer.nmr.mgh.harvard.edu). Hence, the centre of each triangle determined the location of a single current dipole whose orientation was normal to the surface of the triangle.

The temporal subdictionary *G* consists of complex-valued chirplets (chirp functions modulated in amplitude by Gaussian envelopes of different scales) defined as

1 $$\begin{aligned} g_{\gamma }(t)= & {} L(\gamma )\, \exp \left( -\pi \left( \frac{t-\tau }{\sigma } \right) ^{2}\right. \nonumber \\&\left. +\,i \left( \omega \left( t-\tau \right) +\frac{\kappa }{2}\left( t-\tau \right) ^{2}\right) \right) , \end{aligned}$$

where *t* is time, $\gamma =(\tau , \omega , \sigma , \kappa )$, and $L(\gamma )$ is such that $||g_{\gamma }||=1$. Here, $\tau$ denotes translation, i.e. the position of the centre of the Gaussian envelope in time, $\omega$ is the angular frequency, $\sigma$ is the scale corresponding to the temporal span of the Gaussian envelope, $\kappa$ is the angular chirp rate, i.e. the instantaneous rate of change of the frequency, and *i* is the imaginary unit. We opted for chirplets mainly for two reasons: first, the use of chirplets is a more universal approach compared to the sole use of Gabor functions, since the latter constitute a subset of the former. Second, chirplets appear to be more appropriate to parametrize waveforms such as the auditory M100 response, whose onset and offset slopes differ in their steepness. We used a set of chirplets for $N_g$ discrete $\gamma$s and $N_t$ points in time *t*. The upper bounds for the parameters $\omega$ and $\kappa$ were selected such that they sufficiently addressed the frequency spectrum of the M100 waveform. Hence, the temporal subdictionary is an $N_t \times N_g$ matrix, whose columns will be referred to as *temporal atoms*, and it was chosen to be dyadic (see also Mallat and Zhang 1993). Furthermore, due to the linearity of the quasi-static approximation, the morphology of each temporal atom is identical for the sensor and the source space.

### Spatio-temporal matching pursuit (STMP)

Spatio-temporal matching pursuit (STMP) is our generalization of MP into the spatio-temporal domain of multichannel signals; its mathematical description is given by (2) below. Note that it can be performed in the real-valued or complex-valued domain. We decided for the complex form of a chirplet and the analytic representation of a signal achieved by means of the Hilbert transform, owing to the universality and clearness of the underlying equations and the easiness of their implementation. Note also that in the complex-valued approach, the normalization is independent of the chirplet phase.

For a trial-averaged signal *X* being represented by an $N_c \times N_t$ matrix (number of MEG channels $\times$ number of time points), we define the iterative STMP decomposition of *X* into spatio-temporal atoms as^1^

2 $$\begin{aligned} \left\{ \begin{array}{lll} &{} R^{(1)} = H(X), \\ &{} \left( s_d^{(n)}\!,s_g^{(n)}\right) = \displaystyle {\mathop {{\text {argmax}}}\limits _{\begin{array}{c} n_d=1, \ldots , N_d; \ n_g=1, \ldots , N_g \end{array}}\left| \sum _{c=1}^{N_c}\sum _{t=1}^{N_t}D_{n_d,c} R^{(n)}_{c,t} G^{*}_{t,n_g}\right| }\\ &{} = \displaystyle {\mathop {{\text {argmax}}}\limits _{\begin{array}{c} n_d=1, \ldots , N_d; \ n_g=1, \ldots , N_g \end{array}}\left| P_{n_d,n_g}^{(n)}\right| },\\ &{} R^{(n+1)} = R^{(n)} - \left( P_{s_d^{(n)}\!,s_g^{(n)}}^{(n)} G_{:,s_g^{(n)}}D_{s_d^{(n)}\!,:}\right) '\!. \end{array}\right. \end{aligned}$$

Here, $H\!\left( X\right)$ is the discrete-time analytic signal obtained using the Hilbert transform of the trial-averaged signal *X*, computed for each channel *c* separately. $R^{(n)}$ is the residual signal left after subtracting the atoms fitted in the previous iterations, starting from $R^{(1)}$. $\left( s_d^{(n)}\!,s_g^{(n)}\right)$ denotes the indices of the spatial and temporal atoms selected in the *n*th iteration. $P^{(n)} = D R^{(n)} G^{*}$ is the $N_d \times N_g$ matrix to be searched across to find the best fitting *spatio-temporal atom*, i.e. the optimal combination of a spatial and a temporal atom, see Sect. 2.1. Note that the matrix $P^{(n)}$ is complex-valued, i.e. it carries information on both the amplitude and the phase of a selected atom. Furthermore, the energy is conserved exactly as in multichannel matching pursuit, i.e. $||X||^2 = \sum _n |P^{(n)}_{s^{(n)}_d, s^{(n)}_g}|^2 + ||R^{(n+1)}||^2$; see Eq. (17) in Mallat and Zhang (1993).

Because the measured MEG signal—and, hence, its average across trials—is real-valued, the real part of each temporal atom has to be computed as ultimate representation of the reconstruction of the signal by means of that atom at the very end of the decomposition process (2). For the sake of readability, we will occasionally skip the expression “the real part of” whenever its presence is obvious from the context.

### Extension to subdictionaries with very large numbers of elements

Typically, $N_d \approx 15{,}000$ to 25,000 different dipole locations per hemisphere are used in the source analysis of evoked magnetic fields. In the present STMP implementation, we worked with values of $N_d$ covering the range from $\sim$15,000 up to $\sim$600,000. Our intention behind the use of a very large number of locations has been guided by the default value of the FreeSurfer software package which is 300,000 per hemisphere, corresponding to a 1-mm resolution in MRI. However, along with the large number of chirplets used in our computations, $N_g \approx 9 \times 10^4$, and with $N_c = 148$ MEG channels and $N_t = 613$ time samples (length of analysed time interval: 601.62 ms; sampling rate: 1017.25 Hz), the number of operations required to compute $P^{(n)}$ for the largest number of $N_d = 6 \times 10^5$ is then of the order of $N_c\,N_g\,(N_d+N_t)\approx 8 \times 10^{12}$. This causes a significant problem concerning computational resources. Note that the computational complexity of MMP is $\mathcal {O}\left( N_c N_t \log \left( N_t\right) \right)$; however, MMP does not address the source localization aspect. Hence, the overall cost depends on the complexity of the subsequent inverse solution strategy.

Therefore, in order to make the computation feasible even for such a large number of dipole locations, and, hence, the application of STMP more practicable and attractive for the user, we propose not to compute each single element of $P^{(n)}$ but, instead, to apply a procedure based on the Cauchy–Schwarz inequality (Steele 2004). This approach allows disregarding all elements of $P^{(n)}$ whose values are smaller than an adaptively chosen threshold without computing them explicitly. As a result, we end up with a submatrix of $P^{(n)}$, which is small enough to be inspected element-wise. This Cauchy–Schwarz-inequality-based procedure has been successfully applied to the analysis of the two simulations and of the real data presented in this work and will be described in more detail below.

#### Application of the Cauchy–Schwarz inequality

First, we compute the singular value decomposition (SVD) of the trial-averaged data $X=\frac{1}{K}\sum _{k=1}^{K} X^{(k)}$, where $X^{(k)}$ denotes data from the *k*th single trial ($k=1, \ldots , K$). The SVD results in two square matrices, *U* of size $N_c \times N_c$ and *V* of size $N_t \times N_t$, and the $N_c \times N_t$ matrix *S* of zeros but the leading diagonal whose elements, i.e. the singular values, are sorted in descending order. The signal is thus explained by a series of SVD components, where the *j*th component is defined as a triple $\left( S_{j,j},U_{:,j},V_{:,j}\right)$, i.e.

3 $$\begin{aligned} X=U\!SV'=\sum _{j=1}^J S_{j,j}U_{:,j}V_{:,j}', \end{aligned}$$

where $J=\min \left( N_c,N_t\right)$. Once the values of the elements of the leading diagonal of *S* were evaluated, those SVD components that exceed a certain threshold of the signal energy can be selected for further analysis. Next, we replace, without loss of generality, *U* with $U_{:,1:J}, S$ with $S_{1:J,1:J}$, and *V* with $V_{:,1:J}$.^2^

Second, we express $P^{(n)}$ from (2) (for simplicity, we label this matrix *P* for one given iteration) as $P = AB$ with

4 $$\begin{aligned} A= & {} DU\!S^q, \end{aligned}$$

5 $$\begin{aligned} B= & {} S^{1-q} V'G^{*}, \end{aligned}$$

where *A* is of size $N_d \times J$ and *B* of size $J \times N_g$, and *q* is a real-valued parameter discussed later in more detail. Since the overcomplete^3^ spatio-temporal dictionary is designed such that it covers the entire cortex and comprises a huge number of possible waveforms, there will be many spatio-temporal atoms for each iteration of the decomposition of a given signal, whose product with the current signal residuum is small. As a consequence, *P* contains numerous very small, and thus practically irrelevant, elements.

In order to eliminate those elements of *A* and *B* that cannot result in the maximum element of *P*, we propose to make use of the Cauchy–Schwarz inequality (Steele 2004),

6 $$\begin{aligned} \left| \sum _{j=1}^J A_{n_d,j} B_{j,n_g}\right| ^2 \le \sum _{j=1}^J \left| A_{n_d,j}\right| ^2 \sum _{j=1}^J \left| B_{j,n_g}\right| ^2. \end{aligned}$$

Let us define

7 $$\begin{aligned} m_d = \mathop {{\text {argmax}}}\limits _{n_d=1, \ldots , N_d}\sum _{j=1}^J \left| A_{n_d,j}\right| ^2, \end{aligned}$$

8 $$\begin{aligned} m_g = \mathop {{\text {argmax}}}\limits _{n_g=1, \ldots , N_g}\sum _{j=1}^J \left| B_{j,n_g}\right| ^2, \end{aligned}$$

and the threshold $\zeta$ as

9 $$\begin{aligned} \zeta =\max \left( \max _{y}\left| \sum _{j=1}^J A_{m_d,j} B_{j,y}\right| ^2\!, \max _{z}\left| \sum _{j=1}^J A_{z ,j} B_{j,m_g}\right| ^2 \right) \!. \end{aligned}$$

Now we can reject from *A* the elements ($r_d$) that satisfy

10 $$\begin{aligned} \sum _{j=1}^J \left| A_{r_d,j}\right| ^2 \sum _{j=1}^J \left| B_{j,m_g}\right| ^2 < \zeta , \end{aligned}$$

and from *B* those elements ($r_g$) that fulfil

11 $$\begin{aligned} \sum _{j=1}^J \left| A_{m_d,j}\right| ^2 \sum _{j=1}^J \left| B_{j,r_g}\right| ^2 < \zeta . \end{aligned}$$

Hence, we eventually obtain two matrices, $\tilde{A}$ of size $\left( N_d - \#r_d\right) \times J$ with $\#$ denoting the cardinality of a set and $\tilde{B}$ of size $J \times \left( N_g - \#r_g\right)$, that have to be multiplied with each other. Next, we have to search across the resulting matrix to find its maximal absolute element; see (2). This element corresponds to the best fitting spatio-temporal atom, given by the $s_d^{(n)}$th row of *D* and the $s_g^{(n)}$th column of *G*. After a few iterations, $\#r_d$ and $\#r_g$ become small, which increases the dimensions of $\tilde{A}$ and $\tilde{B}$, and, in consequence, the cost of their multiplication. In other words, with consecutive iterations, fewer and fewer elements satisfy Eqs. (10) and (11), hence the gain from the Cauchy–Schwarz “trick” becomes smaller.

The vector $\sum _{j=1}^J \left| A_{r_d,j}\right| ^2$ (and, analogously, $\sum _{j=1}^J \left| B_{j,r_g}\right| ^2$) can be sorted prior to the aforementioned rejection criterion. Finding the first element that satisfies the threshold condition allows rejecting all smaller elements without the need to perform explicit multiplications and related comparisons.

#### The role of *q*

Altering the value of *q* [see (4) and (5)] results in the rejection of different numbers of elements of the matrices *A* and *B*. Note that the sets of indices indicating the rejected elements for different *q*s do not necessarily meet the relation of inclusion. Namely, a *q* that implicates the largest possible number of rejected elements does not have to result in rejecting some of the elements that would be rejected when using another *q*. Our analysis indicates that the number of rejected elements decreases strongly when $q \notin \left[ 0,1\right]$. Since the final results of the STMP decomposition do not depend on the actual value of *q*, our implementation starts with $q = 0.5$, which is a “balanced” choice given the formulation in (4) and (5). The value of *q* is updated by the algorithm if needed, i.e. if the number of elements to be multiplied for a given *q* is still too large, meaning when the number of multiplications to perform in a given STMP iteration is greater than the assumed limiting number. In such a case, *q* can vary by natural multiples of ${\pm }0.1$ within the $\left[ 0,1\right]$ interval. For each *q* value used until the sufficient *q* has been reached, the set of the elements to be rejected is stored, and the sum of those sets defines all the elements to be rejected in the current iteration. If the number of multiplications to be performed in a given iteration is still greater than the limiting number for all 11 values of *q*, the computation will be terminated resulting in $n-1$ spatio-temporal atoms.

In general, one can use matrices $\hat{A} = AC$ and $\hat{B} = C^{-1}B$ instead of *A* and *B*, where *C* is any invertible matrix (a plausible simplification is to use a diagonal invertible *C*). The problem of finding *C* that results in rejecting the largest number of elements of $\hat{A}$ and $\hat{B}$ appears extremely difficult. In the implementation described in this paper, the rejection rates suffice to compute a few iterations, which was adequate for our purposes of estimating a few ECDs.

We would like to emphasize that one can, of course, also use a smaller number of potential dipole locations, and we did so in our simulations addressing the STMP performance for different signal-to-noise ratios; see Sect. 3.1.2. The Cauchy–Schwarz inequality and its implementation is an enhancement of the original STMP approach outlined in Sect. 2.2 in that it enables handling of large dictionaries efficiently. Hence, it completes the essential part of this work, i.e. the novel concept of a spatio-temporal dictionary (Sect. 2.1) and the resulting strategy of *simultaneous* decomposition of the data in space *and* time (Sect. 2.2).

## Application of STMP to simulated and real MEG data

### Simulations

To examine the efficiency of the STMP algorithm, we carried out simulations. We tested its performance in two scenarios, where STMP was compared with (1) two other multichannel-MP (MMP) approaches (Sect. 3.1.1), and (2) a non-MMP approach (Sect. 3.1.2). In both cases, ECDs were seeded at distinct locations of the brain, and the resulting magnetic fields were superimposed with noise from real MEG data. Furthermore, in order to test a specific localization property of the algorithm—i.e. its capability to separate two ECDs with different waveforms but identical location—we performed additional simulations without noise (Sect. 3.1.3). Note that we did not apply noise in the latter simulations on purpose, since only then one is able to evaluate the properties of the algorithm itself as opposed to assess its *performance* at, for example, several SNRs.

#### Performance of STMP in comparison with MMP approaches

**Fig. 1:**
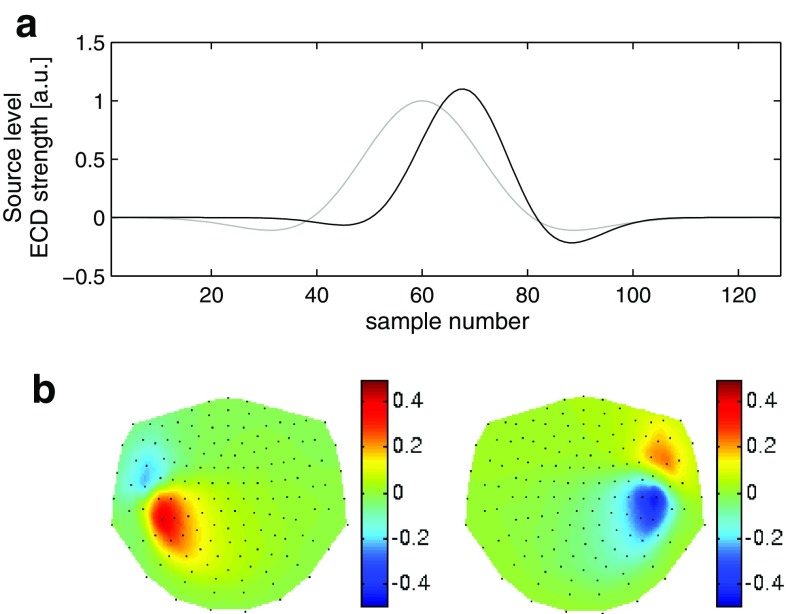
Simulated signal. **a** Time courses of the two simulated ECDs. The *grey curve* shows the source waveform of the ECD seeded in the left auditory cortex, and the *black curve* the ECD seeded in the right auditory cortex. The scaling of the ordinate was deliberately chosen as (time) samples to reflect the generality of the approach. **b** Reconstructed spatial magnetic field pattern at the peak latencies of the two waveforms shown in (**a**). Nasion is on top, left ear to the left. The *black dots* represent the MEG channels

We opted for simulated data coarsely mimicking measurements from an auditory experiment to conform to the application of STMP to the selected real data as outlined in the subsequent section (Sect. 3.2). For this purpose, in each hemisphere one ECD was seeded at a distinct location of the polygon triangulation grid (see Sect. 2.1) using the MR image of an individual subject’s brain. The [*x*, *y*, *z*] coordinates of these two ECDs (in cm) in a Cartesian coordinate system spanned by the three fiducials^4^ were [0.38, 6.26, 4.36] for the ECD in the left hemisphere and $[2.00, -6.19, 4.45]$ for the ECD in the right hemisphere. The waveforms of these two ECDs were Gabor functions of slightly different scales, frequencies, translations, phases, and peak amplitudes (Fig. 1a). Figure 1b shows the resulting dipolar magnetic field patterns generated by each ECD at its respective peak latency. The magnetic fields of the two ECDs were superimposed with noise taken from the baseline period of trial-averaged real MEG data. The overall SNR of the simulated multichannel signal was set to ${\sim }83$ estimated from the real data with respect to the M100 waveform (see Sect. 3.2).

**Fig. 2:**
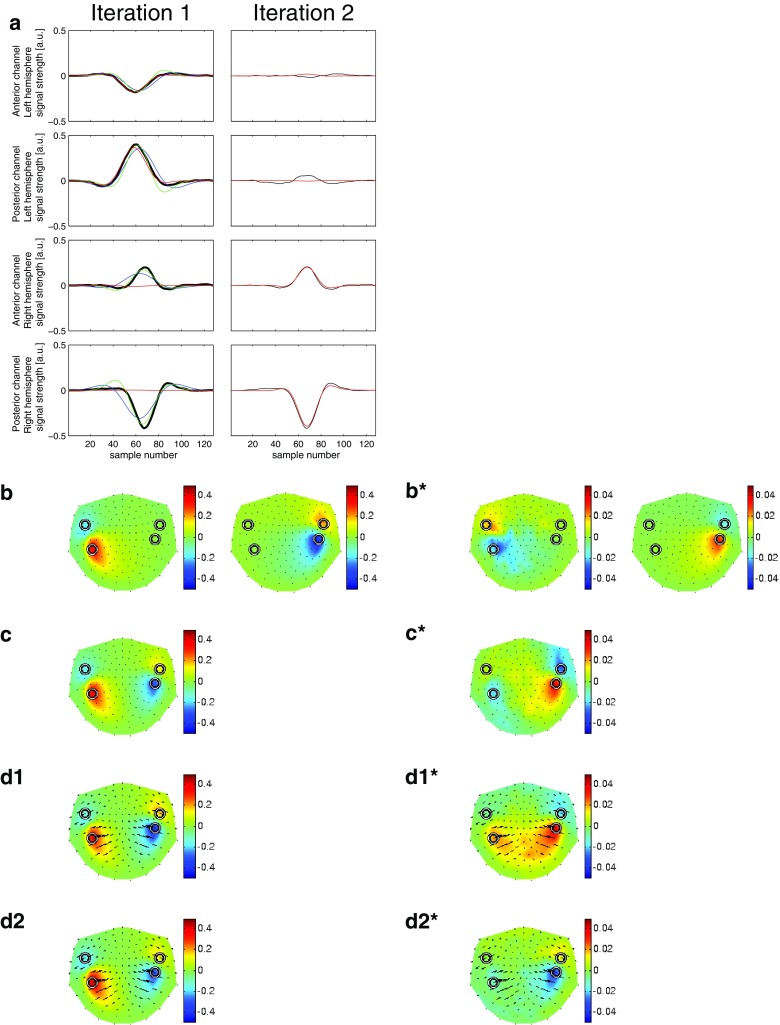
Comparison of STMP (see Sect. 2.2) versus MMP (Durka et al. 2005; Matysiak et al. 2005) using simulated signals. **a** The *panels in the two top rows* show data from the two MEG channels with the largest signal above the left hemisphere, the *panels in the two bottom rows* from the two MEG channels with the largest signal above the right hemisphere. The *first and third rows* represent the anterior and the *second and fourth rows* the posterior channels. The *panels of the left column* display the simulated signal (*thick black curves*), along with the real part of the complex-valued waveforms resulting from the first iteration obtained by means of STMP (*red curves*). For comparison, results of constant-phase MMP (Durka et al. 2005) (*blue curves*) and varying-phase MMP (Matysiak et al. 2005) (*green curves*) are also shown. The *panels of the right column* display the real part of the residua (*thin black curves*) resulting from the first STMP iteration and the waveforms of the second iteration of STMP only (*red curves*). **b** Reconstructed fields of the ECDs of the two STMP iterations at the peak latencies of the corresponding waveforms in (**a**). Nasion is on top, left ear to the left. The *circles* depict the locations of the four selected sensors in (**a**). **c** Like (**b**) but for constant-phase MMP, at the single peak latency of the corresponding waveforms in (**a**). **d1, d2** Like (**b**) but for varying-phase MMP, at peak latencies of the ECDs shown in Fig. 1. The *length of the arrows* corresponds to the modulus of the complex amplitudes of the fitted Gabor functions, and their *orientation* reflects the phase. The *panels marked with an asterisk* depict the difference between their counterparts on the left and the simulated field (Fig. 1). Note that the *scale on the colour bar of the asterisk-marked panels* is an order of magnitude smaller

Figure 2 shows a comparison of the decomposition of the simulated signal for three different MP approaches: STMP and two MMP versions, viz. the constant-phase MMP (Durka et al. 2005) and the varying-phase MMP (Matysiak et al. 2005). In Fig. 2a, the left column displays the results of the first iteration for all three approaches, whereas the right column displays the result of the second iteration of the STMP decomposition only. The two panels on top of each column refer to the two MEG channels with the largest negative (first row, at an anterior location) and the largest positive (second row, at a posterior location) peak amplitude above the left hemisphere. The two panels at the bottom refer to the two MEG channels with the largest positive (third row, at an anterior location) and the largest negative (fourth row, at a posterior location) peak amplitude above the right hemisphere. These selected channels can be identified as circles in Fig. 2b–d, which display, for STMP (b), constant-phase MMP (c), and varying-phase MMP (d1, d2), a projection of the 3D-layout of all MEG channels (small black dots) onto a plane. Note that the algorithms were applied to all 148 MEG channels simultaneously and that only four selected channels are shown for reasons of clarity.

The simulated waveforms at these four channels (thick black curves in the panels of the left column of Fig. 2a) depict the solution to the forward problem based on the source waveforms of Fig. 1a. Their peak magnitudes (in a.u.) are, from top to bottom: $-0.18_{(59)}, 0.40_{(60)}, 0.20_{(67)}, -0.42_{(67)}$, where the subscripts show the corresponding peak latencies (in samples). The waveforms computed for all 148 channels constitute the input to the first iteration of the three approaches.

The first STMP iteration results in a spatio-temporal atom related to the peak waveforms of the left hemisphere only. The real part of the corresponding temporal atom is depicted by the red curves and well accounts for the morphology of these waveforms (two left top panels of Fig. 2a). The peak magnitudes and latencies of these waveforms are $-0.19_{(58)}$ and $0.39_{(58)}$, in close concordance with the simulated data ($-0.18_{(59)}$, $0.40_{(60)}$). The resulting waveforms above the right hemisphere (red curves in the two left bottom panels of Fig. 2a) are close to zero throughout the entire time interval. The black curves in iteration 2 of Fig. 2a are the residua obtained after subtracting the STMP parametrization of iteration 1. Their overall magnitudes are very small at the two top channels, but remain almost unchanged compared to the original waveform at the two bottom channels.

The second STMP iteration (red curves in Fig. 2a, right column) provides the spatio-temporal atom which well fits the peak waveform of the channels above the right hemisphere. Peak magnitudes and latencies are $0.20_{(67)}$ and $-0.40_{(67)}$, again in striking concordance with the simulated data ($0.20_{(67)}, -0.42_{(67)}$). Clearly, these two atoms address the activity of the two ECDs separately and well reflect their different waveforms, peak magnitudes, and peak latencies. This separability of the two ECDs is characteristic of the STMP algorithm. It is convincingly illustrated in Fig. 2b, where, for each of the two STMP iterations, the reconstructed magnetic field is shown, i.e. the real part of the product of: (1) the forward field from the spatial subdictionary *D*, (2) the corresponding chirplet from the temporal subdictionary *G*, and (3) the complex multichannel weight (see Sect. 2.2), at the peak latency of the product of the last two of the three.

The [*x*, *y*, *z*] coordinates of the location of the two spatial atoms of the STMP decomposition were [0.22, 6.37, 4.32] for the atom resulting from the first iteration, i.e. for the ECD in the left hemisphere, and $[2.00, -6.19, 4.45]$ for the atom of the second iteration, i.e. for the right hemisphere. The source location obtained for the right hemisphere exactly matches the location of the seeded ECD. For the left hemisphere, the STMP decomposition resulted in a small deviation between seeded and computed source location. The distance between these two locations was 2 mm.

In contrast to STMP, the two MMP parametrizations address the activities of the left and the right ECD simultaneously, i.e. within one (the first) iteration. This can be inferred from the panels of the first iteration of Fig. 2a, and from the respective reconstructed field distributions in Fig. 2c, d. Figure 2a also displays, in addition to the temporal atoms of the STMP decomposition, the Gabor functions obtained from the constant-phase (blue curve) and varying-phase MMP (green curve). For the constant-phase MMP, the decomposition results in a single atom describing the waveforms of the ECDs of two hemispheres. Consequently, compared to STMP, constant-phase MMP provides an inferior compromise whenever the time courses of individual ECDs differ from each other, and, hence, it cannot account for the different peak latencies generated by the two sources. The corresponding peak magnitudes and latencies obtained for the four waveforms were $-0.16_{(63)}, 0.35_{(63)}, 0.13_{(63)}, -0.31_{(63)}$. Figure 2c shows the respective reconstructed magnetic field at the peak latency of the selected atom. Notably, the field pattern above the right hemisphere is less spread out than that obtained with STMP (Fig. 2b).

The release of the phase constraint in the varying-phase MMP decomposition results in a very good match of the waveforms of the temporal atoms to the simulated waveforms for the channels above the left hemisphere, but less so for the right hemisphere (Fig. 2a). The peak magnitudes and latencies were $-0.18_{(61)}, 0.39_{(62)}, 0.17_{(66)}, -0.37_{(66)}$. Figure 2d1, d2 displays the respective result of the varying-phase MMP at peak latencies of the ECDs shown in Fig. 1.

The panels on the right, marked with an asterisk, show the difference between the results shown on the left and the simulated field (Fig. 1). Note that the scale of the colour bars of the asterisk-marked panels is an order of magnitude smaller than that in the panels on the left side.

The field maps in Fig. 2d show the real-valued reconstruction, i.e. the real part of the complex-valued (Matysiak et al. 2005) representation. The length of the ticks corresponds to the modulus of the complex amplitudes of the fitted Gabor functions, and their orientation reflects the phase. For a better comparison, the peak magnitudes and latencies of the simulated and reconstructed waveforms are summarized in Table 1.

The computation of the energy of the residua provides meaningful information concerning the similarity of the waveforms between the simulated and reconstructed data for the three approaches. The respective values for the waveforms of the four channels displayed in Fig. 2a are $\{0.02, 0.08, 0.04, 0.06\}$ for the two iterations of STMP, $\{0.05, 0.20, 0.17, 0.46\}$ for the first iteration of constant-phase MMP, and $\{0.06, 0.22, 0.04, 0.14\}$ for the first iteration of varying-phase MMP. These values support the findings shown in Fig. 2a, according to which STMP outperforms the two MMP approaches. However, one should bare in mind that it is difficult to define an objective criterion for such judgements because, in principle, the intrinsic greediness of MP does not necessarily have to address the features of interest in the best (whatever this would mean) possible way.

#### Comparison with non-MMP approaches

In general, the comparison of one source localization approach with another one is a critical and non-trivial issue, since, from a mathematical point of view, the question concerning the superiority of one inverse-problem method over another is, in principle, ill-posed. This issue becomes even more complicated when the inverse solution of a discrete (dipolar) approach like STMP ought to be compared with that of a distributed approach. Thus, we restrict ourselves to a comparison of STMP with another discrete approach, the well-established RAP-MUSIC algorithm (Mosher and Leahy 1999) using the Python implementation (Gramfort et al. 2013a, 2014) from http://martinos.org/mne. We computed the localization error for the two simulated atoms (Fig. 1) for 28 different SNRs, where we defined SNR as the square of the ratio of the largest singular values—one for the signal in the nominator and one for the noise in the denominator—of a trial-averaged multichannel time course arranged as channels $\times$ samples. Noise was taken from the pre-stimulus baseline of a real MEG data set comprising 190 single trials (see also Sect. 3.2.1). In order to obtain a realistic scenario, two subsets of baseline epochs (noise) were generated, one used to generate simulated noise in both approaches (STMP and RAP-MUSIC), and one to produce the noise estimate additionally required by RAP-MUSIC only. These two subsets had to differ from each other, since otherwise RAP-MUSIC would have 100% information about the noise. Thus, from the entire set of baseline epochs, we randomly selected and averaged 95 for each of 1000 realizations of the simulated noise, allowing for repeated selection. The remaining 95 baseline epochs were used to compute the noise estimate required by the RAP-MUSIC algorithm. Note also that the waveforms of the two simulated ECDs (Fig. 1) are highly coherent, which makes this scenario very challenging for any inverse solution method. To make the computational cost of these extensive simulations manageable, we used $\sim$15,000 potential dipole locations.

**Table 1:** Simulated and reconstructed peak magnitudes and latencies of Fig. 2a

Selected channel	Peak magnitude			
	Simulated	Reconstructed		
		STMP	cpMMP	vpMMP
Anterior left	$-$0.18	$-$0.19	$-$0.16	$-$0.18
Posterior left	0.40	0.39	0.35	0.39
Anterior right	0.20	0.20	0.13	0.17
Posterior right	$-$0.42	$-$0.40	$-$0.31	$-$0.37

Selected channel	Peak latency			
	Simulated	Reconstructed		
		STMP	cpMMP	vpMMP
Anterior left	59	58	63	61
Posterior left	60	58	63	62
Anterior right	67	67	63	66
Posterior right	67	67	63	66

STMP outperforms the two MMP approaches, i.e. the constant-phase (cp) and the varying-phase (vp) MMP, in all but two comparisons of simulated with reconstructed peak magnitudes. Only the peak magnitudes derived for the varying-phase MMP waveforms of the anterior and posterior left channels show a slightly better match to the simulated data than the waveforms derived by means of STMP. The STMP peak latencies almost perfectly match the simulated data. Here, STMP also outperforms the two MMP approaches in all comparisons

**Fig. 3:**
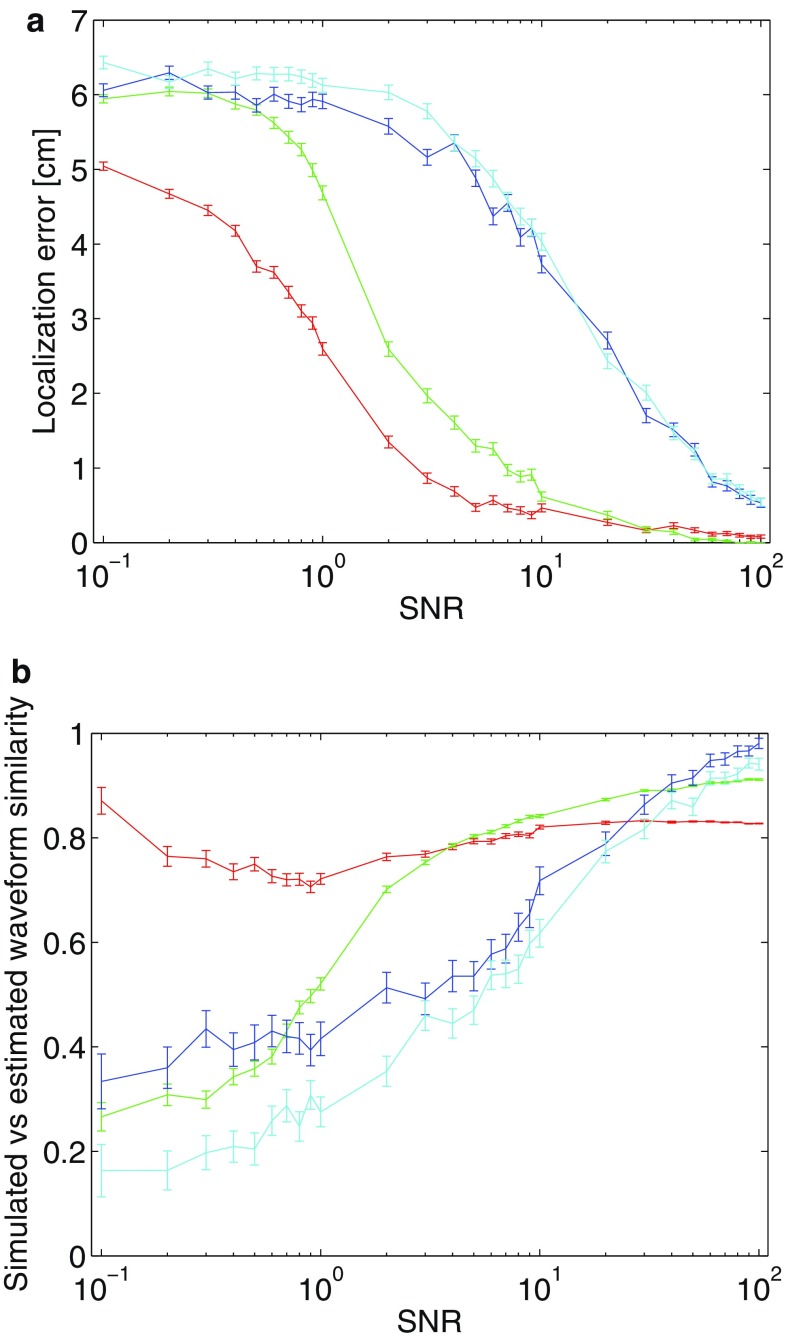
Performance of STMP (see Sect. 2.2) versus RAP-MUSIC (Mosher and Leahy 1999) for various SNRs. The two methods are compared for the two simulated ECDs from Fig. 1, for a series of SNRs. For a detailed description of the simulations, see Sect. 3.1.2. **a** Localization error in cm. **b** Similarity of the estimated and the simulated waveform; the value 1 indicates a perfect match. *Colour coding* for both panels: 1st STMP atom (*red*), 2nd STMP atom (*green*), 1st RAP-MUSIC solution (*blue*), 2nd RAP-MUSIC solution (*cyan*). The *bars* depict the measurement uncertainty originating from 1000 realizations of the noise for each SNR

**Fig. 4:**
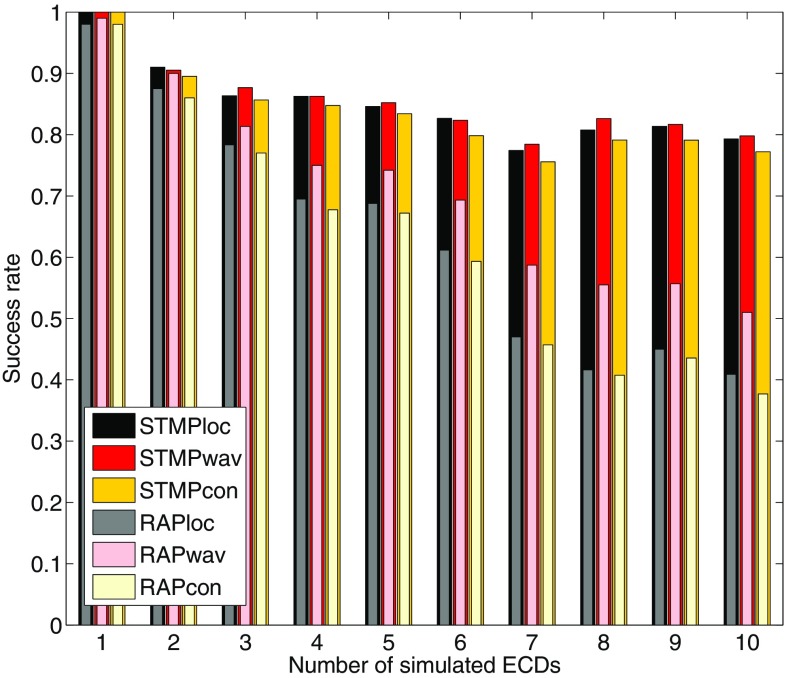
Performance of STMP (see Sect. 2.2) versus RAP-MUSIC (Mosher and Leahy 1999) for simulations with different numbers of ECDs. Normalized success rate of finding the location (*loc*, *first bar in a three*), the waveform morphology (*wav*, *second bar*), and both (*con*, *third bar*)—i.e. logical conjunction for the location and the morphology—correctly for STMP (*dark colours*) versus RAP-MUSIC (*light colours*). 100 random realizations were used (Sect. 3.1.2)

Figure 3 shows the results of the localization error (panel a) and of the similarity analysis of the estimated and the simulated waveforms (panel b) of the two ECDs obtained with STMP (red and green curves) and RAP-MUSIC (blue and cyan curves). The signal-to-noise ratio covers three orders of magnitude, from 0.1 to 100. We found for all SNRs that the localization error obtained with STMP was smaller than that obtained with RAP-MUSIC (Fig. 3a). This result is most pronounced for the first ECD (red curve) in the entire SNR range, and for the second ECD (green curve) in the range between about 1 and 100. The vertical bars depict the measurement uncertainty originating from the 1000 realizations. Figure 3b shows, for each of the two estimated ECDs, the similarity of the estimated and the simulated ECD waveform, defined as the ratio $\frac{\text {wav}_{\text {sim}} \cdot \text {wav}_{\text {est}}'}{\text {wav}_{\text {sim}} \cdot \text {wav}_{\text {sim}}'}$, where $\text {wav}_{\text {sim}}$ denotes the simulated and $\text {wav}_{\text {est}}$ the estimated waveform; the products are scalar. For the first ECD, STMP outperforms RAP-MUSIC for SNR values up to 20; for larger SNRs, RAP-MUSIC provides slightly higher similarity values than STMP. For the second ECD, STMP outperforms RAP-MUSIC for SNR values up to 50. For SNRs of 60 and 70, the two approaches provide statistically indistinguishable solutions. Only for the largest SNRs of 80, 90 and 100, RAP-MUSIC performs better.

In a further simulation, we investigated how STMP and RAP-MUSIC performed when multiple ECDs had to be estimated. Up to 10 ECDs were randomly seeded in the cortex, and their time courses were randomly assigned to elements of the temporal subdictionary. The minimum distance between any pair of seeded ECDs was 2 cm, and their maximum similarity (same measure as above) was 0.8. Each of such 10 configurations was realized 100 times. Noise was generated and added like in the aforementioned simulations. The SNR value was again set to 83 taken from the simulation described in Sect. 3.1.1. We then compared the performances of STMP and RAP-MUSIC in their ability to reconstruct the locations and time courses of the simulated ECDs. The triple bars in Fig. 4 reflect the normalized success rate in finding the location (first bar in a triple), the morphology (second bar), and both (third bar)—i.e. logical conjunction for the location and the morphology—correctly. The decision threshold for whether or not an estimation was correct was set to 0.5 cm for the distance between the seeded and the localized ECD location, and to 0.9 for the similarity between simulated and estimated waveform. For all numbers of simulated ECDs, STMP clearly outperforms RAP-MUSIC.

#### Separability of components using STMP

We also performed simulations (probabilistic test) in order to address another aspect of the performance of the algorithm, its ability to disentangle two temporal components originating from the same spatial location, in more detail. We simulated two ECDs which were located at the same point in space, but differed in their waveforms. The location—one out of about 15,000—and the two waveforms were chosen randomly. In total, we performed 100 random realizations. We found that, in 71% of all cases, the two waveforms were localized at exactly the same—and correct—location. 90% of all cases fell within a 1-cm error margin, and 99% within 2 cm. There was only one single case, out of 100, with a large localization error of about 6 cm, which may have resulted from one of those difficult locations in the cortex where most likely all localization methods fail. These simulations were deliberately performed without noise. Results might change, of course, if noise was added, but then the possibly negative effect would be due to the noise, whereas the idea behind noise-free simulations was to show that the algorithm itself is mathematically capable of separating two waveforms at the same location. Furthermore, one should bear in mind that some of the randomly chosen locations may have had an unfavourable distance to, and/or orientation with respect to, the sensors. In summary, the results of this simulation regarding the STMP performance are convincing, despite the ill-posedness of the inverse problem.

### Real data

#### STMP applied to evoked magnetic fields from an auditory MEG experiment

To test the performance of STMP on real MEG data, we applied the algorithm to auditory evoked magnetic fields that originated from an MEG measurement with a subject who was exposed to a repeated binaural stimulation with a 500-ms long 1-kHz sinusoidal tone presented at a sound pressure level of 90 dB. The tone was repeated 224 times, with a stimulus onset interval of 2 s. Magnetic fields were acquired using a 148-channel whole-head magnetometer MEG system (Magnes 2500, 4D-Neuroimaging), with a sampling rate of 1017.25 Hz. Preprocessing of the data (notably artefact rejection due to, for example, eye blinks) reduced the number of trials used in the final analysis to 190.

**Fig. 5:**
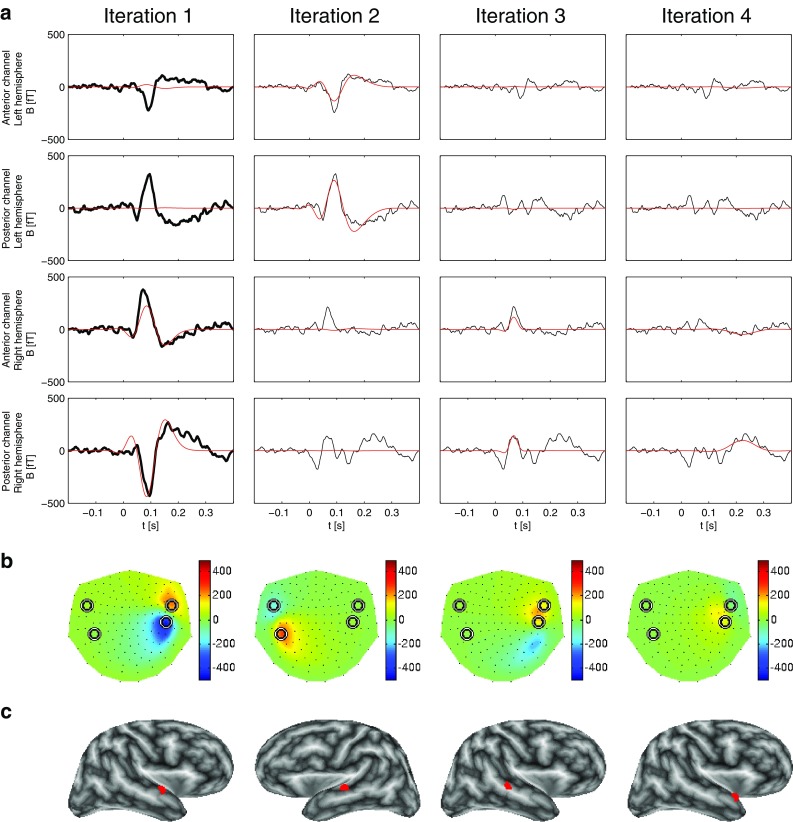
Results of the STMP decomposition (see Sect. 2.2) of real data. **a** The *four columns* show the first four STMP iterations. The *two top rows* display the two MEG channels with the largest magnetic field *B* above the left hemisphere, the *two bottom rows* the two channels with the largest *B* above the right hemisphere. The *first and third rows* represent the anterior and the *second and fourth rows* the posterior maximum channels. The *thick black curves* in the panels of iteration 1 represent the original trial-averaged MEG signals. The *thin black curves* in the panels of iterations 2–4 represent the real part of the complex residua of the previous iteration. The *red curves in each panel* depict the real part of the fitted waveforms, i.e. of the temporal atoms from the subdictionary *G*. **b** Reconstructed forward field pattern (in [fT]) of the ECDs of the four iterations at the peak latencies of the corresponding waveforms in (a). Nasion is on top, left ear to the left. The *circles* depict the locations of the four selected sensors of (**a**). **c** The corresponding ECD locations (*red dots*) are embedded in the inflated representation of the subject’s brain. The ECDs of iterations 1 and 4 were localized in the STG of the right hemisphere, the ECD of iteration 2 in the STG of the left hemisphere. The ECD derived in the third iteration was located on the bank of STS of the right hemisphere (see Sect. 3.2)

Figure 5a displays the result of the STMP decomposition of the trial-averaged AEFs on the sensor level. The four columns show the results from the first four iterations. Similarly to Fig. 2a, for each of these four iterations, the two rows of panels on top show the waveforms of the two MEG channels with the largest negative (at an anterior location) and the largest positive (at a posterior location) peak amplitude, i.e. the auditory M100 waveform, in the left hemisphere, and the reverse pattern of the right hemisphere is displayed in the panels of the two bottom rows. The time window used in the analysis ranged from −200 to 400 ms and encompasses the prestimulus baseline interval (from −200 ms to stimulus onset at $t=0$ ms) and the entire M100 component.

The thick black curves in each panel of the first column of Fig. 5a represent the trial-averaged AEFs measured at the location of the respective MEG channel, whose analytic representations—see (2)—are to be decomposed, for all 148 channels simultaneously, in the first iteration. The thin black curves in the panels of the successive columns depict the real part of the signal to be decomposed in the successive iterations, i.e. of the residua of the preceding iteration. The red curves in all panels are the reconstructions obtained from the STMP algorithm; they are the best match to the average AEF (first iteration) or the residuum in consecutive iterations. They depict the real part of the product of the iteration-specific temporal atom (chirplet) and the corresponding complex-valued scaling coefficient adjusting the amplitude and phase.

The first iteration results in a spatio-temporal atom related to the M100 waveform of the right hemisphere, as indicated by the dipolar field pattern of the corresponding spatial atom in the first panel of Fig. 5b. The corresponding real part of the chirplet, i.e. of the temporal atom obtained in this iteration, well accounts for the morphology of the waveforms of the corresponding MEG channels (two bottom panels in Fig. 5a); it explains 38.5% of the total signal energy. Its magnitude at the two channels above the opposite, left hemisphere (red curves in the two top panels in Fig. 5a) is very small. The second iteration provides the spatio-temporal atom describing the M100 component above the left hemisphere; see second panel of Fig. 5b and the first two panels of the second iteration of Fig. 5a. The energy of this atom is 18.5%, about half of that of the atom of the first iteration. This finding nicely reflects this subject’s characteristics of the M100, which shows a clear hemispheric asymmetry of the magnetic field strength, with the dominance above the right hemisphere. Iterations 3 and 4 both provide atoms with much smaller energy, i.e. 5.9 and 6.1%, respectively. We set the decomposition to stop as soon as the energy of an atom in the consecutive iteration would reach the value smaller than 5% of the energy of the original signal. Since this was the case for atom number 5 (3.15%), we restrict the representation to the first four atoms.

**Fig. 6:**
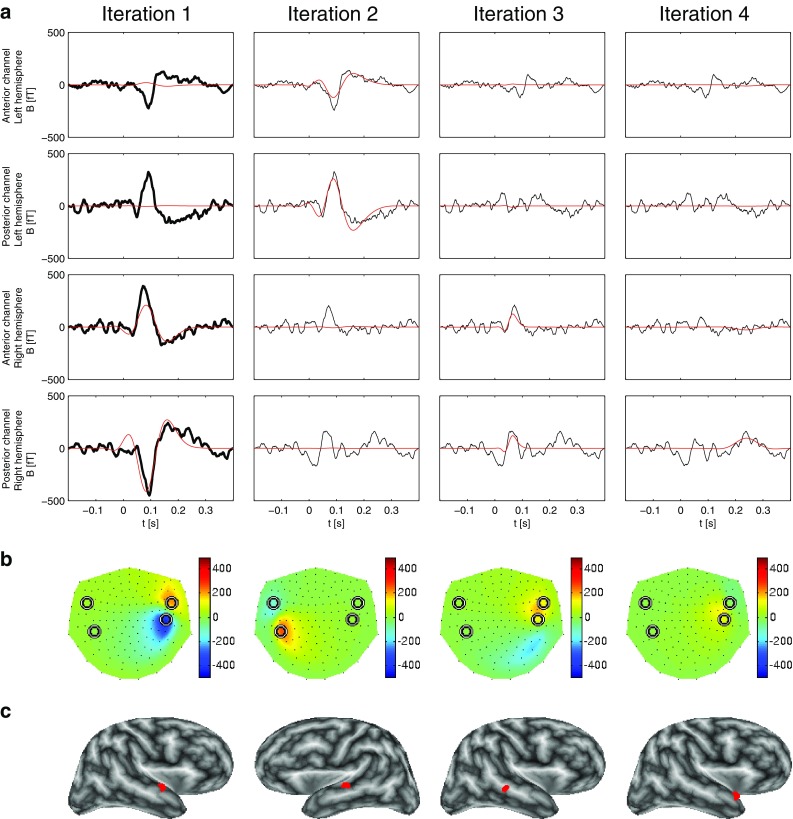
Results for STMP decomposition of a trial-averaged signal from 95 trials

**Fig. 7:**
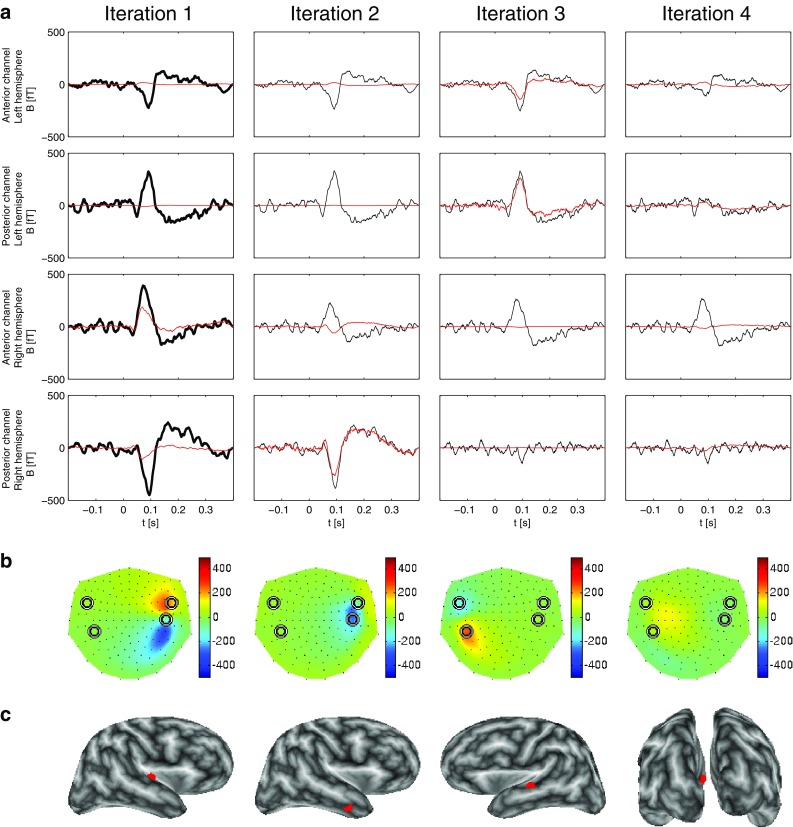
Results obtained with RAP-MUSIC applied to the trial-averaged signal from 95 trials

**Fig. 8:**
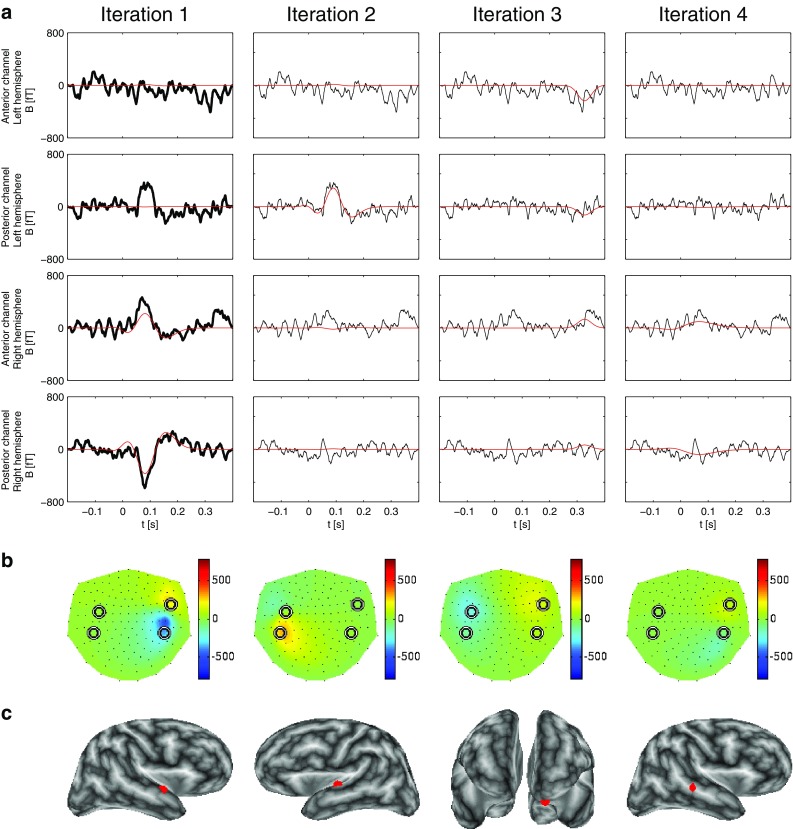
STMP for 12 trials, i.e. every 16th trial from the original dataset of 190 trials. Note that at this SNR the maximum absolute signal was found at slightly different channels compared to the data with larger SNRs

**Fig. 9:**
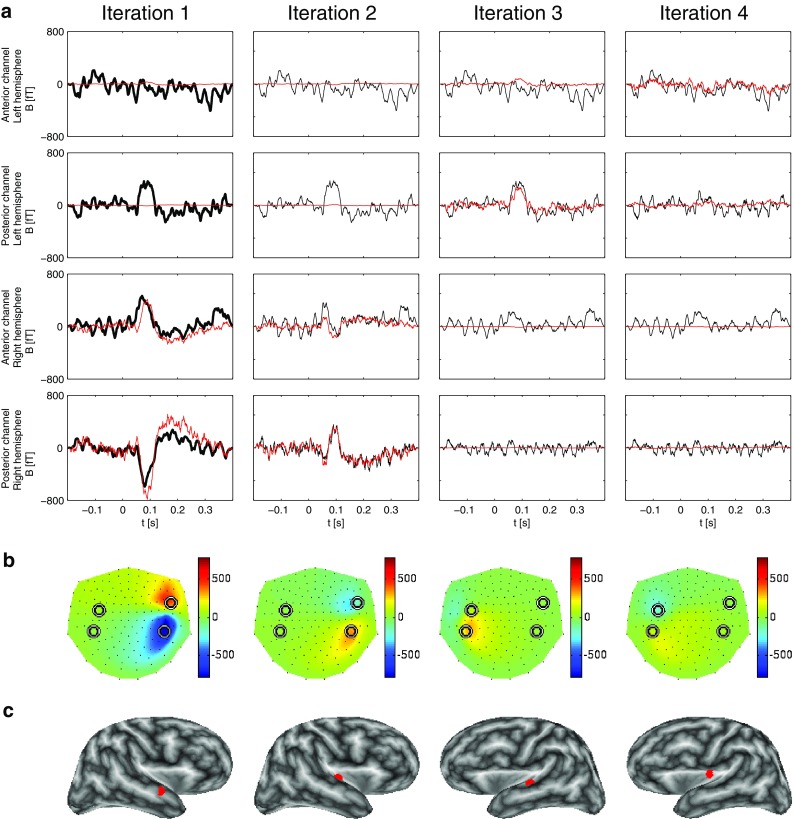
RAP-MUSIC for 12 trials, i.e. every 16th trial from the original dataset of 190 trials. Note that at this SNR the maximum absolute signal was found at slightly different channels compared to the data with larger SNRs

Figure 5c shows the locations of the four ECDs represented by the spatial atoms of the decomposition. All panels show an inflated brain with the white matter surface computed using the FreeSurfer software, based on the subject’s individual MRI scan and coded in grey scale according to the local curvature. The ECDs of iterations 1 and 4 were localized in the superior temporal gyrus (STG) of the right hemisphere, the ECD of iteration 2 in the STG of the left hemisphere. The ECD derived in the third iteration was located on the bank of the superior temporal sulcus (STS) of the right hemisphere.

#### STMP versus RAP-MUSIC on real data

For a comparison of STMP with RAP-MUSIC on real data, we analysed the same MEG data introduced in Sect. 3.2.1 at several SNRs. Since RAP-MUSIC requires an independent estimate of the noise (see Sect. 3.1.2), we divided the original set of 190 trials into two sets of 95 trials each. The trials with odd numbers were used to derive the signal of interest (the mean across those trials), whereas trials with even numbers served as basis for noise estimation. The noise estimate was obtained by subtracting the mean across odd trials from the even single trials. In this approach, noise is estimated within a time window whose length is equal to that of the signal to be analysed. In general, 95 trials are sufficient to provide a good SNR in a simple auditory experiment like the one considered here.

Figure 6 shows the results from STMP on the subset of averaged 95 trials. They are very similar to those for the entire set of 190 trials. Figure 7 depicts the corresponding results for RAP-MUSIC. These results are somewhat different, with the first two iterations explaining the right-hemispheric activity, and the third iteration addressing the left auditory source. Given the localization of the source in the second iteration and the split of the right-hemispheric M100 into two very distinct components, we tend to argue that this result is rather less convincing than that of STMP. The fourth RAP-MUSIC iteration describes some non-auditory activity.

We continued with further dividing the original set of 190 trials by integer multiples of two, i.e. by taking every 4th, 8th, and 16th trial for the average signal to be examined, and so, respectively, for the noise estimate. These resulting subsets consisted of 48, 24, and eventually as few as 12 trials. For 48 trials (see Figs. 1 and 2 in Supplementary material), STMP well described the AEFs in both hemispheres, whereas RAP-MUSIC failed to explain the left auditory-cortex activity. For 24 trials (Figs. 3 and 4 in Supplementary material), the STMP results remained consistent with those obtained from larger SNRs. RAP-MUSIC addressed the left auditory cortex well, yet the quality of source localization in the right hemisphere worsened. For as few as 12 trials, the STMP results still remained fairly stable (Fig. 8), whereas RAP-MUSIC overfitted heavily in the first iteration (Fig. 9) resulting in a large error introduced to iteration 2 and, thus, a spurious source. The third iteration, however, handled the left-hemisphere activity very well. The analysis for even smaller SNRs turned out to be pointless. The SNR was simply too small to provide sufficient information for the two algorithms.

Overall, our analysis of real data shows the superiority of STMP compared to RAP-MUSIC especially at small SNRs. Moreover, another strong observation is the consistency of the STMP results across a wide range of SNRs, from 190 down to 12 trials. This is also in line with the findings presented in the following Sect. 3.2.3.

#### Robustness of the STMP algorithm

**Fig. 10:**
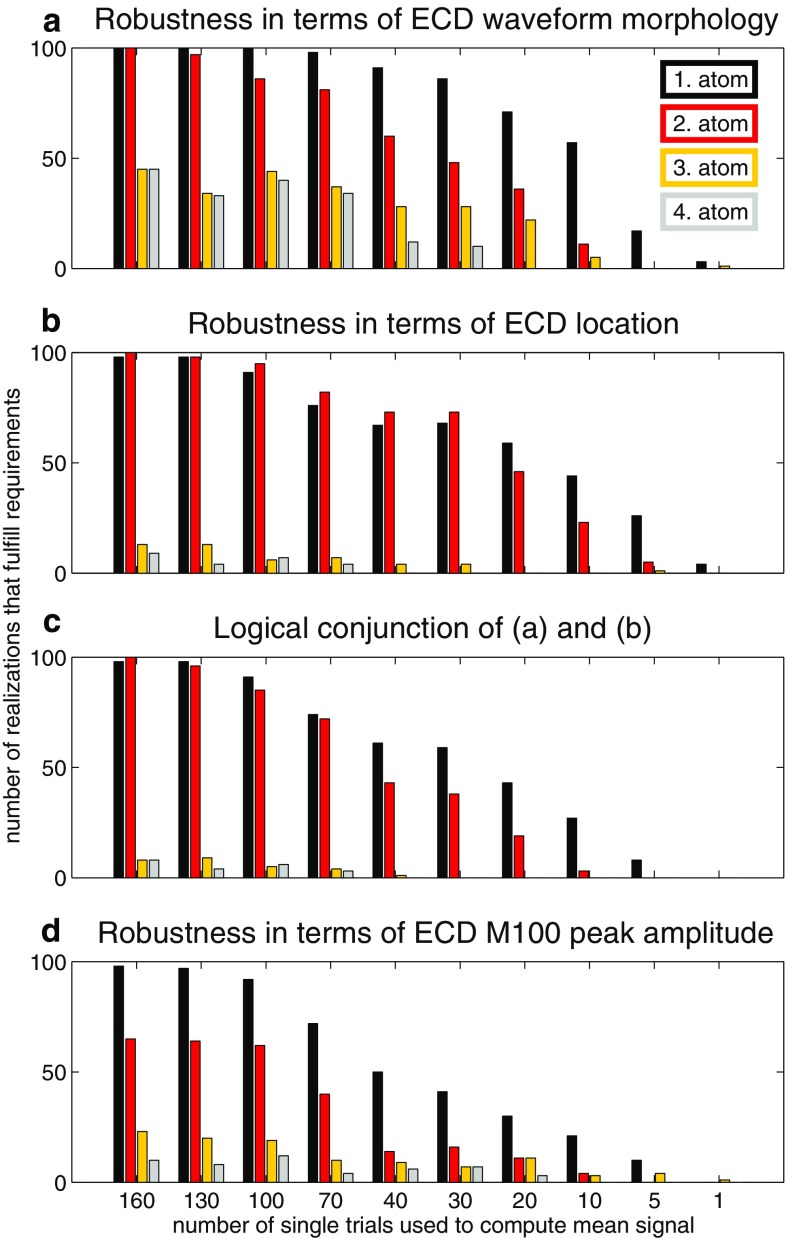
Performance of the STMP algorithm (see Sect. 2.2) with subsets of different trials numbers. *Each panel* shows the number of realizations that fulfil the following requirements obtained for a comparison of the different subsets with the original data set of 190 trials: **a** the normalized product of ECD time courses is larger than or equal to 0.9, **b** the spatial Euclidean distance of the estimated locations is smaller than or equal to 5 mm, **c** the logical conjunction of (**a**) and (**b**), and **d** the ratio of the peak amplitudes of the reconstructed ECD waveforms lies within the [0.95, 1.05] interval

In order to further check the robustness of the STMP algorithm, we compared the results of the decomposition of the trial-averaged signal of the original (full) real data set of 190 trials with those for the average signals derived from smaller subsets of different sizes, viz. 160, 130, 100, 70, 40, 30, 20, 10, 5, and 1 trial(s). We randomly selected the trials for these subsets from all 190 trials such that no single trial was selected multiple times for a subset, and then performed the STMP decomposition of the trial-averaged signal of each subset. The random selection process was performed 100 times for each subset size in order to reduce a possible bias that might otherwise emerge from trials having a larger/smaller SNR than others. Hence, the number of subsets was 1,000, i.e. 100 for each of the ten sizes. The advantage of this approach is that it provides information on the performance of the algorithm based on characteristics of real single-trial data, not on specific assumptions concerning, for example, signal and/or noise characteristics of the data as it would be the case in simulations. Figure 10 shows the results of the comparison of the decomposition of the original data set with those of the various subset sizes for three different measures. In all panels, the abscissa indicates the number of single trials of each subset size, and the ordinate the number of realizations that fulfil the particular requirement of the respective measure.

Figure 10a provides information on the similarity of the morphology of the source waveforms (ECD time courses), i.e. the real part of the temporal atoms, between the original data and the subsets of different sizes. We used, as similarity measure, the normalized product between the waveform obtained for the full data set and the waveforms estimated for the subsets, evaluated separately for each of the four ECDs. The height of a bar denotes the number of random realizations for which that product was larger than or equal to 0.9. Figure 10a indicates that for the atoms of the first two iterations, i.e. the two atoms which represent the main component of the M100 in each hemisphere, the decomposition provides robust results down to a subset of 70 trials, which is about 37% of the total number of 190 trials. Even for subsets of 20 trials $\sim$75 realizations of the first atom and $\sim$45 realizations of the second atom still fulfilled these requirements. Compared to the first two atoms, atom 3 and atom 4 show a similar trend as to the dependence of the number of realizations from the subset size, yet with clearly smaller values of realizations.

Figure 10b provides information on the robustness of the source localization, i.e. of its reproducibility when subsets of fewer and fewer trials are decomposed. The bars in this figure indicate the number of realizations for which the spatial Euclidean distance between the estimated source location obtained for the original data set of 190 trials and that for subsets of trials was smaller than or equal to 5 mm. The results obtained for the first spatial atom resemble the findings shown in Fig. 10a for the temporal atom of the first iteration; only for subsets that contain less than $\sim$70 the number of realizations starts to decrease from 75%. Interestingly, the number of successful realizations obtained for the second atom is equal to or larger than the corresponding number for the first atom for the first six of the ten subset sizes. This finding indicates that greediness of MP algorithms is less of a concern in the spatial domain of STMP.

Figure 10c shows the logical conjunction of the thresholding conditions addressed in panels (a) and (b), i.e. the number of realizations for which the two aforementioned conditions were met. This figure shows the quality of agreement in terms of ECD spatial locations and the morphology of their time courses. Down to the subset size of 70 trials, it closely resembles the results of the robustness of the source localization shown in Fig. 10b.

In Fig. 10d, the reproducibility of the algorithm with regard to the reconstruction of the M100-peak amplitude of the reconstructed waveforms (temporal atoms) for the different data sets is addressed. For each subset size, the ordinate indicates the number of realizations whose ratios between the respective peak amplitudes fell within the [0.95, 1.05] interval. Compared to the analyses in Fig. 10a–c, the M100-peak amplitude of the first atom is relatively stable, but less so for the second atom, which may stem from the aforementioned weaker left-hemispheric response in this subject.

**Fig. 11:**
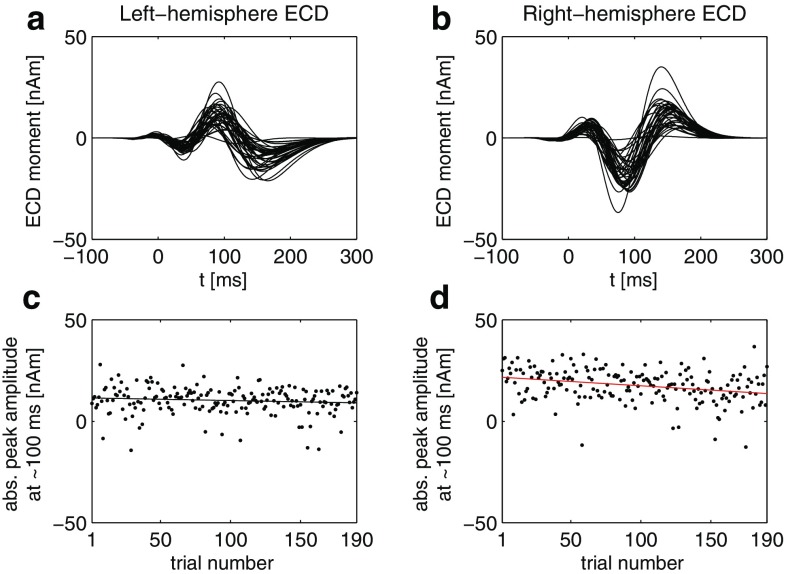
Projection onto single trials. Single-trial waveforms of ECDs obtained from projecting the results of the first two STMP iterations (see Sect. 2.2) onto single trials for the left (**a**) and right (**b**) hemisphere. For the sake of clarity, only every fifth trial is shown. The absolute values of the corresponding reconstructed M100-peak amplitudes across consecutive trials are shown in (**c**) and (**d**). The *solid lines* in (**c**) and (**d**) are fits of a first-degree polynomial to the data. The associated slopes are $-0.013$ with $\mathrm {CI}_{95} = [-0.030, 0.005]$ for the left-hemisphere ECD (c), and $-0.042$ with the 95% confidence interval $\mathrm {CI}_{95} = [-0.063, -0.022]$ for the right-hemisphere ECD (d). The *red colour* of the fitted line in (**d**) indicates that the slope is significantly different from zero

#### Single-trial analysis based on STMP

The procedure outlined in Sect. 2.2 describes the linear expansion of trial-averaged data into *N* spatio-temporal atoms. These atoms can be further projected onto the *K* single trials, and, thus, also allow studying the trial-to-trial variation of the signal. This results in $K \times N$ time series of the dipolar moments. Following Sielużycki et al. (2009b), we incorporate a single-trial model,

12 $$\begin{aligned} X_{c,t}^{(k)} = \mathrm {Re} \sum _{n=1}^{N} \beta ^{(k,n)}\,W_{c,t}^{(n)} + \varepsilon _{c,t}^{(k)}, \end{aligned}$$

where the data $X^{(k)}$ in trial *k*, which origin from $N_c$ channels and $N_t$ time moments, are represented as the real part of the sum of $W^{(n)}=d^{(n)}\times g^{(n)}$ weighted by the trial-specific coefficient $\beta ^{(k,n)}$, and the contaminating stochastic noise $\varepsilon ^{(k)}$. The column vector $d^{(n)}$ is an element of the spatial subdictionary *D* and reflects the location of the *n*th activity in space, whereas the row vector $g^{(n)}$ is an element of the temporal subdictionary *G* and reflects the corresponding waveform. Such a projection of the atoms onto single trials can be seen as a space–time–frequency filter obtained adaptively from the decomposition of the trial-averaged signal.

Figure 11a, b shows projections of the temporal atoms obtained from the first two STMP iterations for the trial-averaged real data, i.e. for the sources located in the left (a) and right (b) auditory cortex (see Fig. 5), onto single trials according to the approach from Sielużycki et al. (2009b); see also Eq. (12). For the sake of clarity, only every fifth trial is shown. Both figures display a trial-dependent peak latency at around 100 ms, corresponding to the single-trial latency variation of the auditory M100 (Sielużycki et al. 2009b). Figure 11c, d shows the corresponding trial-to-trial variation of the reconstructed M100-peak amplitude for these two atoms, along with the slope of the fitted first-degree polynomial to the single-trial peak amplitudes. The slope differed significantly from zero for the source located in the right hemisphere (d); its value was $-0.042$, 95% confidence interval $\mathrm {CI}_{95} = [-0.063, -0.022]$. For the source in the left hemisphere (c), the slope was $-0.013, \mathrm {CI}_{95} = [-0.030, 0.005]$. The reconstructed single-trial latency did not reveal a significant change across trials, in neither of the two hemispheres.

## STMP: an advanced approach in the analysis of MEG data

We developed a new MP-based algorithm that enables *simultaneously*, within each iteration, finding a solution to the inverse problem in space and parametrizing the characteristics of multichannel MEG waveforms in the time–frequency domain, with the important advantage of a sparse signal representation both in space and time. The core of the STMP algorithm—and the novelty of this approach—is a dictionary composed of a spatial and a temporal subdictionary, with dipolar fields reflecting the spatial and chirplets reflecting the temporal characteristics of the components of the MEG signal. Note that the choice of a particular dictionary is only a matter of the assumptions behind the experiment. The algorithm itself, and our Matlab implementation in particular, do not impose any constraints in this matter.

The three pivotal assumptions of the STMP approach are: (1) the underlying brain activity is focal, which justifies the use of the ECD source model, (2) the time-varying signal of an ECD can be well described by a chirplet, and (3) spatio-temporal characteristics of ECDs underlying the measured data can be identified sequentially, one after another, in an iterative manner. We have demonstrated superiority of this algorithm over two previous MP-based approaches (Durka et al. 2005; Matysiak et al. 2005) by means of simulations. When applied to illustrative MEG data acquired with a simple auditory paradigm, we found that STMP outperforms RAP-MUSIC especially at small SNRs. The decomposition provided convincing temporal atoms to represent the MEG time courses and plausible loci for the ECDs underlying the M100 waveforms.

### Simultaneous STMP versus sequential approaches

The proposed approach offers an important advantage over previous two-step approaches (see, e.g., Durka et al. 2005; Matysiak et al. 2005; Gratkowski et al. 2008; Lelic et al. 2009, 2011) in which, first, waveforms on the sensor level are decomposed into temporal (time–frequency) atoms, and, second, selected atoms are then used to solve the inverse problem. Note that we intentionally refrained in our simulations from performing ECD source localization after decomposing the data by means of constant-phase or varying-phase MMP. Unlike for STMP, in which source localization is an inherent property, the two MMP approaches should in this context be considered as preprocessing stages which require selecting a particular inverse solution method—among many existing strategies, with potentially different outcomes. Thus, it appeared justified to us not to arbitrarily select one specific inverse solution approach for a comparison with STMP. Furthermore, Gramfort et al. (2013b) emphasized in their discussion that estimation errors made in the first step are crucial as to the accuracy of the source estimates, since the first step, i.e. the decomposition of the waveform, does not take into account the solution of the inverse problem. The STMP approach overcomes this problem, since it simultaneously provides a spatio-temporal solution for each iteration. Since space constitutes an inherent property of the dictionary, each ECD in this approach has its own temporal morphology independent of those of other ECDs.

Contrary to many discrete dipole fitting approaches, the proposed STMP algorithm is free of any a priori assumptions concerning the number of underlying sources. In contrast to various distributed source solutions, STMP is free of constraints otherwise imposed by, for example, regularization parameters (see Gramfort et al. 2013b, and references therein). STMP is a source localization strategy in which each signal component of the decomposition, i.e. each spatio-temporal atom, represents the activity of exactly one dipolar source. This means that STMP is able to effectively separate brain activities (ECDs) and to assign specific waveforms to each of them even if they differ in only one of the four aspects (space, time, frequency, scale), which also makes greediness of MP less of a concern. Furthermore, the elements of the spatial and temporal subdictionaries can be readily adjusted to specific time intervals or spatial regions of interest. In this way, STMP circumvents such intricate inverse solutions distinctive for classical MP preprocessing approaches which are restricted to a temporal dictionary only and where the decomposition of a time-varying signal (which may contain contributions of temporally related but spatially distinct brain activities) yields one sole waveform addressing two or more activities.

The auditory evoked M100 waveforms generated in the left and the right hemisphere are a simple and intelligible example for this scenario. They could be well reconstructed by means of the first two STMP iterations (Fig. 5). The temporal characteristics of the third atom matched the time course of the residual features in the right hemisphere that seem to reflect the non-perfect match to the M100 component in the first iteration. Along with the spatial location of this atom, this finding indicates that the M100 waveform may emerge from multiple sources (see, e.g., Lütkenhöner et al. 2003; Lütkenhöner 2003). Iteration 4 addressed a later component. We confine ourselves to assign a meaningful interpretation to the results of the first two iterations only, to avoid arbitrary judgements on what may be deeply buried in noise (König et al. 2007).

The observation of a habituation effect in the single-trial analysis of the M100-peak amplitude, i.e. the decrease of the brain response to repeated stimuli over consecutive trials, is in accordance with the results reported by Sielużycki et al. (2009a) and Sielużycki et al. (2009b) for the same data set. Note, however, the differences between this and the two previous approaches: in the current one, the habituation effect can be assigned to the sources, unlike in Sielużycki et al. (2009a, b), where its observation was restricted to the sensor level.

### Performance of the STMP algorithm

The overall very good performance of the STMP algorithm, concerning the reproducibility of the results as well as the handling of noise, is convincingly demonstrated in Fig. 10. The larger the number of single trials in a subset, the larger the number of realizations that fulfil the respective requirements. Note that the results of the analysis of the entire time courses and estimated locations of the ECDs (Fig. 10a–c) indicate that the number of single trials may be reduced from 190 to 100, or even 70, without a significant deterioration of performance—this notably applies to the first and second atom, i.e. those which have the largest energy and are, usually, in the focus of interest in the analysis of the data. This result may even find its way into the planning of the timing of MEG experiments, since a smaller number of trials allows releasing constraints on other stimulus parameters (for example stimulus duration) or on the stimulus presentation (for example stimulus onset interval).

STMP proves to be remarkably efficient and robust for small and moderate signal-to-noise ratios (see Fig. 3 for simulated as well as Fig. 8 and supplementary Figs. 1 and 3 for real data). These findings strongly indicate that STMP might be particularly useful in those experimental paradigms in which the number of trials must be kept small, for example in experiments with particular time constraints like in clinical settings. In our opinion, the somewhat inferior performance of STMP, in comparison to RAP-MUSIC, in the ability to restore the simulated time courses for the very large SNRs is a relatively little price to pay for the clearly improved spatial localization. Note also that the error bars in Fig. 3 (especially in the similarity analysis shown in the bottom panel) are smaller for STMP compared to RAP-MUSIC, which means that the STMP estimator is more efficient (it has smaller variance) despite being more biased at very large SNRs. However, the size of this effect may potentially stem from the constraints imposed by the size of the temporal dictionary, an aspect which is not applicable to RAP-MUSIC; hence, we would refrain from overemphasising its significance. Another positive feature of STMP is that it is capable of efficiently disentangling multiple ECDs, both in terms of locations and the corresponding time courses (Fig. 4).

We would also like to emphasize here, that, when real data are parametrized, *every* method introduces a certain bias because no model describes the real world perfectly. This is exactly the reason to opt for maximum consistency in simulations. The fact that we simulated the ECD activity with functions from the MP dictionary is not a bias, but, on the contrary, it is mere consistency which enables avoiding bias. Note that the eigenvectors in RAP-MUSIC are unconstrained. They could, in principle, perfectly match the simulated signal—if the SNR allows for it. In fact, one can see clearly from our simulations (Fig. 3) that for large SNRs, the performance of RAP-MUSIC is superior to that of STMP as to the parametrization of the waveforms, but STMP is better in localization. The use of chirplets in this particular application of STMP does not have to work to the advantage of STMP under all circumstances. This constraint is a blessing or a curse, depending on the actual SNR. For simplicity, let us consider the morphology aspect here only. Imagine two cost functions, cost(SNR), on a plane *x*–*y*, where *x* is SNR and *y* is the cost which stems from adaptivity (i.e. lack of constraints). The cost function for STMP increases with increasing SNR, whereas the cost function for RAP-MUSIC decreases. There is a point on the plane where the two functions cross each other. This point corresponds to the SNR at which is does not make a difference whether STMP or RAP-MUSIC is used.

### The problem of computational cost

In order to make the computations feasible in a reasonable amount of time even for subdictionaries with a large number of elements, we suggested to use the Cauchy–Schwarz inequality. However, with consecutive iterations of the decomposition, the residua become less and less “sharp” in terms of their spatio-temporal characteristics. The fact that, at later iterations, we then observe very similar dot products (see (2)) computed for a large number of atoms and the current residuum, is a strong indication that there are no significant spatio-temporal structures in the signal any more, which provides a straightforward stopping criterion, because proceeding any further would only result in solutions that can be considered as negligible. This makes the use of the Cauchy–Schwarz inequality less and less efficient and so increases the computational cost. This, in turn, imposes limitations on the overall number of iterations that can be computed in an acceptable amount of time if at all, as time is not the only limiting factor; RAM capacity of a computer, for example, is by no means less important. Nevertheless, a powerful machine can readily compute the first few iterations. With 16 CPUs and 64 GB of RAM, it took us only a few minutes, as the use of the Cauchy–Schwarz inequality reduced computational cost by 99.1% up to even 99.99998%, depending on iteration.

The problem of computational cost is a common and crucial issue of advanced and precise source localization techniques. It was also addressed recently by Babadi et al. (2014), who proposed the Subspace Pursuit-based Iterative Greedy Hierarchical (SPIGH) solution, in which the cortex is recursively subdivided into smaller and smaller patches. This approach proved to be computationally efficient; see also an earlier work by Obregon-Henao et al. (2012). Future improvements of the STMP algorithm may address combinations of Gabor functions (see Sielużycki et al. 2009a; Gramfort et al. 2013b) or chirplets to model more complex signal morphologies, and/or combinations of several locations characterized by the same morphology to model more complex, e.g., coherent, source configurations. One may also include the entire brain rather than only the cortex as the solution space. Eventually, we plan to extend the approach to combine both the MEG and EEG modality simultaneously.

### Outlook: decomposition of induced signals

As an outlook we would like to mention that we have recently conceptualized an STMP-based approach to the estimation of induced rather than evoked activity by extending the selection criterion from Eq. (2) to

13 $$\begin{aligned} \displaystyle {\mathop {{\text {argmax}}}\limits _{\begin{array}{c} n_d=1, \ldots , N_d; \ n_g=1, \ldots , N_g \end{array}} \sum _{k=1}^K{\left| \sum _{c=1}^{N_c}\sum _{t=1}^{N_t}D_{n_d,c} R^{(n)}_{k,c,t} G^{*}_{t,n_g}\right| }}, \end{aligned}$$

where $k = 1 \dots K$ denotes trial index. Because of the increased dimensionality, this approach implies huge computational costs. However, early analyses with the data from a complex experimental paradigm to study gamma activity are promising. Yet, since they are part of a currently realized project, they are beyond the scope of this paper. Here we only want to signal the possibility of applying STMP to the estimation of the sources of induced activity. Details of technical implementation will be revealed in a future publication.

The Matlab code used for the simulations and real-data analysis, as well as the illustrative MEG data set presented in this work, is available on reader’s request.

## Electronic supplementary material

Below is the link to the electronic supplementary material.

Supplementary material 1 (pdf 520 KB)

## Footnotes

1
$G_{:,l}$ denotes the *l*th column of matrix *G*, and analogously for other matrices; $\mathrm {Re}$ denotes the real part of a complex expression; $G^*$ is the complex conjugate of *G*, and $(\cdot )'$ denotes the transpose of $(\cdot )$.

2 Here, $U_{:,1:J}$ denotes the matrix constructed from the 1st to *J*th columns of matrix *U* and all its rows, and identically so for *V*, while $S_{1:J,1:J}$ is the matrix constructed from the 1st to *J*th columns and rows 1 to *J* of *S*.

3 Overcompleteness means that the dictionary contains more elements than would normally be necessary to fully explain the signal with, e.g., a full orthonormal basis. This usually, and intentionally, results in fewer atoms being needed to explain the sought features of interest.

4
*y*-axis through left and right preauricular points and positive to the left; *x*-axis through nasion and positive to the front; *z*-axis positive to the top.

## References

[CR1] Babadi B, Obregon-Henao G, Lamus C, Hämäläinen MS, Brown EN, Purdon PL (2014). A subspace pursuit-based iterative greedy hierarchical solution to the neuromagnetic inverse problem. NeuroImage.

[CR2] Darvas F, Pantazis D, Kucukaltun-Yildirim E, Leahy RM (2004). Mapping human brain function with MEG and EEG: methods and validation. NeuroImage.

[CR3] Davis G (1994) Adaptive nonlinear approximations. Ph.D. dissertation, New York University, New York

[CR4] Durka PJ, Matysiak A, Martínez-Montes E, Valdés-Sosa P, Blinowska KJ (2005). Multichannel matching pursuit and EEG inverse solutions. J Neurosci Methods.

[CR5] Geva AB (1996) Spatio-temporal mathing pursuit (SToMP) for multiple source estimation of evoked potentials. In: IEEE proceedings of the nineteenth convention of the electronics engineering, pp 113–116

[CR6] Gorodnitsky IF, George JS, Rao BD (1995). Neuromagnetic source imaging with FOCUSS: a recursive weighted minimum norm algorithm. Electroencephalogr Clin Neurophysiol.

[CR7] Gramfort A, Luessi M, Larson E, Engemann D, Strohmeier D, Brodbeck C, Goj R, Jas M, Brooks T, Parkkonen L, Hämäläinen M (2013). MEG and EEG data analysis with MNE-Python. Front Neurosci.

[CR8] Gramfort A, Strohmeier D, Haueisen J, Hämäläinen MS, Kowalski M (2013). Time-frequency mixed-norm estimates: sparse M/EEG imaging with non-stationary source activations. NeuroImage.

[CR9] Gramfort A, Luessi M, Larson E, Engemann D, Strohmeier D, Brodbeck C, Parkkonen L, Hämäläinen M (2014). MNE software for processing MEG and EEG data. NeuroImage.

[CR10] Gratkowski M, Haueisen J, Arendt-Nielsen L, Chen AC, Zanow F (2008). Decomposition of biomedical signals in spatial and time-frequency modes. Methods Inf Med.

[CR11] Hämäläinen M, Hari R, Ilmoniemi RJ, Knuutila J, Lounasmaa OV (1993). Magnetoencephalography—theory, instrumentation, and applications to non-invasive studies of the working human brain. Rev Modern Phys.

[CR12] König R, Sielużycki C, Durka PJ (2007). Tiny signals from the human brain: Acquisition and processing of biomagnetic fields in magnetoencephalography. J Low Temp Phys.

[CR13] Lelic D, Gratkowski M, Valeriani M, Arendt-Nielsen L, Drewes AM (2009). Inverse modeling on decomposed electroencephalographic data: a way forward?. J Clin Neurophysiol.

[CR14] Lelic D, Gratkowski M, Hennings K, Drewes AM (2011). Multichannel matching pursuit validation and clustering—a simulation and empirical study. J Neurosci Methods.

[CR15] Lütkenhöner B (2003) Single-dipole analyses of the N100m are not suitable for characterizing the cortical representation of pitch. Audiol Neurotol 8(4):222–233 10.1159/000071062 12811003

[CR16] Lütkenhöner B, Krumbholz K, Seither-Preisler A (2003). Studies of tonotopy based on wave N100 of the auditory evoked field are problematic. NeuroImage.

[CR17] Mallat S, Zhang Z (1993). Matching pursuits with time-frequency dictionaries. IEEE Trans Signal Process.

[CR18] Matysiak A, Durka PJ, Martínez-Montes E, Barwiński M, Zwoliński P, Roszkowski M, Blinowska KJ (2005). Time-frequency-space localization of epileptic EEG oscillations. Acta Neurobiol Exp.

[CR19] Mosher JC, Leahy RM (1999). Source localization using recursively applied and projected (RAP) MUSIC. IEEE Trans Signal Process.

[CR20] Nolte G (2003). The magnetic lead field theorem in the quasi-static approximation and its use for magnetoencephalography forward calculation in realistic volume conductors. Phys Med Biol.

[CR21] Obregon-Henao G, Babadi B, Lamus C, Brown EN, Purdon PL (2012) A fast iterative greedy algorithm for MEG source localization. In: 2012 annual international conference of the IEEE. Engineering in Medicine and Biology Society (EMBC). IEEE, pp 6748–6751 10.1109/EMBC.2012.6347543 23367478

[CR22] Oostenveld R, Fries P, Maris E, Schoffelen JM (2011). FieldTrip: Open source software for advanced analysis of MEG, EEG, and invasive electrophysiological data. Comput Intell Neurosci.

[CR23] Schomer DL, Lopes da Silva FH (2010). Niedermeyer’s electroencephalography: basic principles, clinical applications, and related fields.

[CR24] Ségonne F, Dale AM, Busa E, Glessner M, Salat D, Hahn HK, Fischl B (2004). A hybrid approach to the skull stripping problem in MRI. NeuroImage.

[CR25] Sielużycki C, König R, Matysiak A, Kuś R, Ircha D, Durka PJ (2009). Single-trial evoked brain responses modeled by multivariate matching pursuit. IEEE Trans Biomed Eng.

[CR26] Sielużycki C, Kuś R, Matysiak A, Durka PJ, König R (2009). Multivariate matching pursuit in the analysis of single-trial latency of the auditory M100 acquired with MEG. Int J Bioelectromagn.

[CR27] Steele JM (2004) The Cauchy–Schwarz master class: an introduction to the art of mathematical inequalities. Cambridge University Press. ISBN: 13 978-0521546775

[CR28] Wendel K, Väisänen O, Malmivuo J, Gencer NG, Vanrumste B, Durka P, Magjarević R, Supek S, Pascu ML, Fontenelle H, Grave de Peralta Menendez R (2009) EEG/MEG source imaging: methods, challenges, and open issues. Comput Intell Neurosci. Article ID 656092. doi:10.1155/2009/656092 10.1155/2009/656092 PMC2715569 19639045

[CR29] Wu SC, Swindlehurst AL (2013). Matching pursuit and source deflation for sparse EEG/MEG dipole moment estimation. IEEE Trans Biomed Eng.

