# Supplementary material for: Simultaneous spatio-temporal matching pursuit decomposition of evoked brain responses in MEG
Source: Biol Cybern. 2017 Jan 21;111(1):69–89. doi: 10.1007/s00422-016-0707-5 (PMC5326632; doi:10.1007/s00422-016-0707-5)
Supplement: Supplementary file 1 — Supplementary material 1 (pdf 520 KB) [file 422_2016_707_MOESM1_ESM.pdf]

Supplementary material to the article

Simultaneous spatio-temporal matching pursuit decomposition of evoked brain responses in MEG

(doi: 10.1007/s00422-016-0707-5)

Paweł Kordowski, Artur Matysiak, Reinhard König, and Cezary Sielużycki

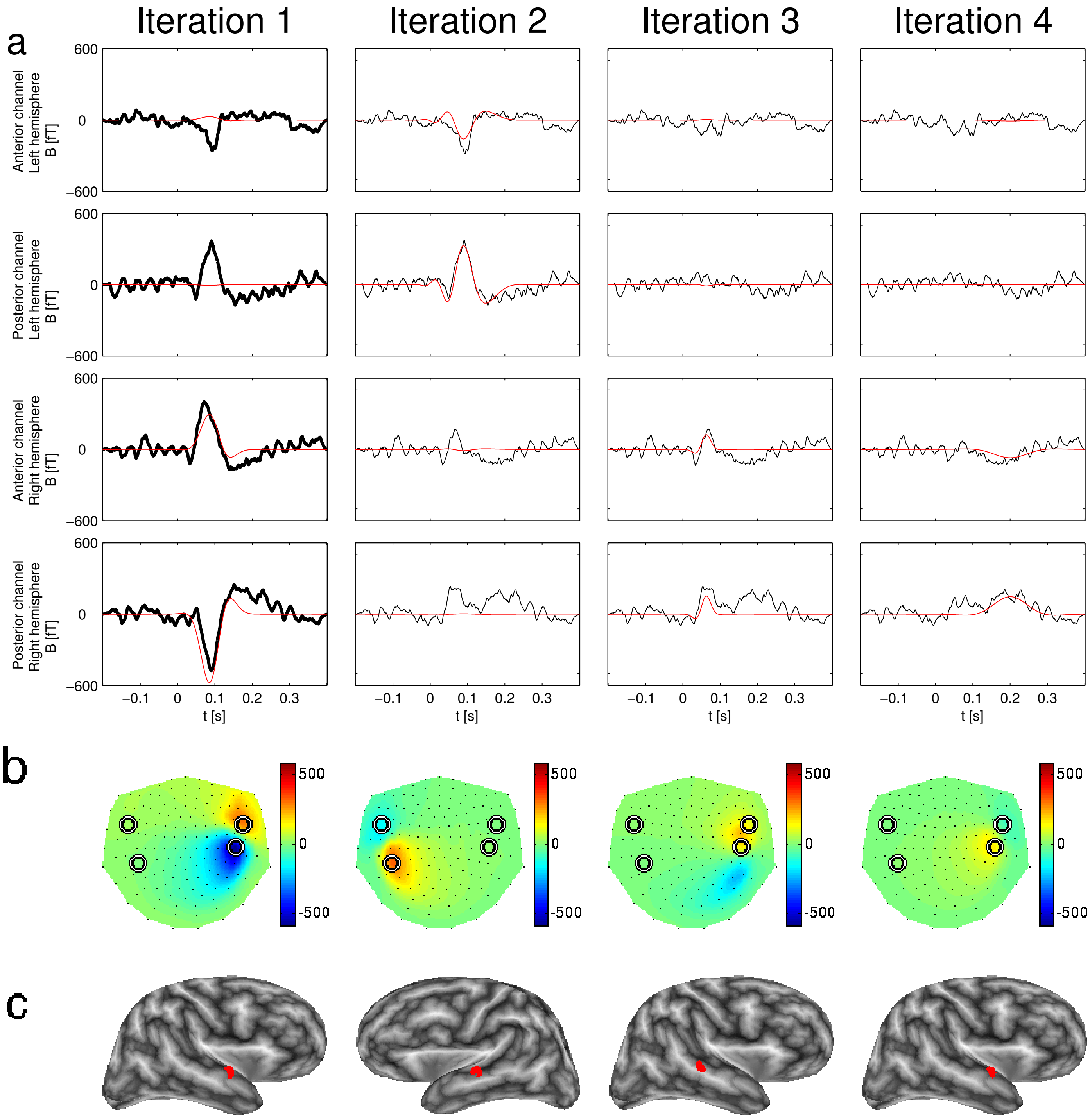

Figure 1: STMP for 48 trials, i.e. every fourth trial from the original dataset of 190 trials.

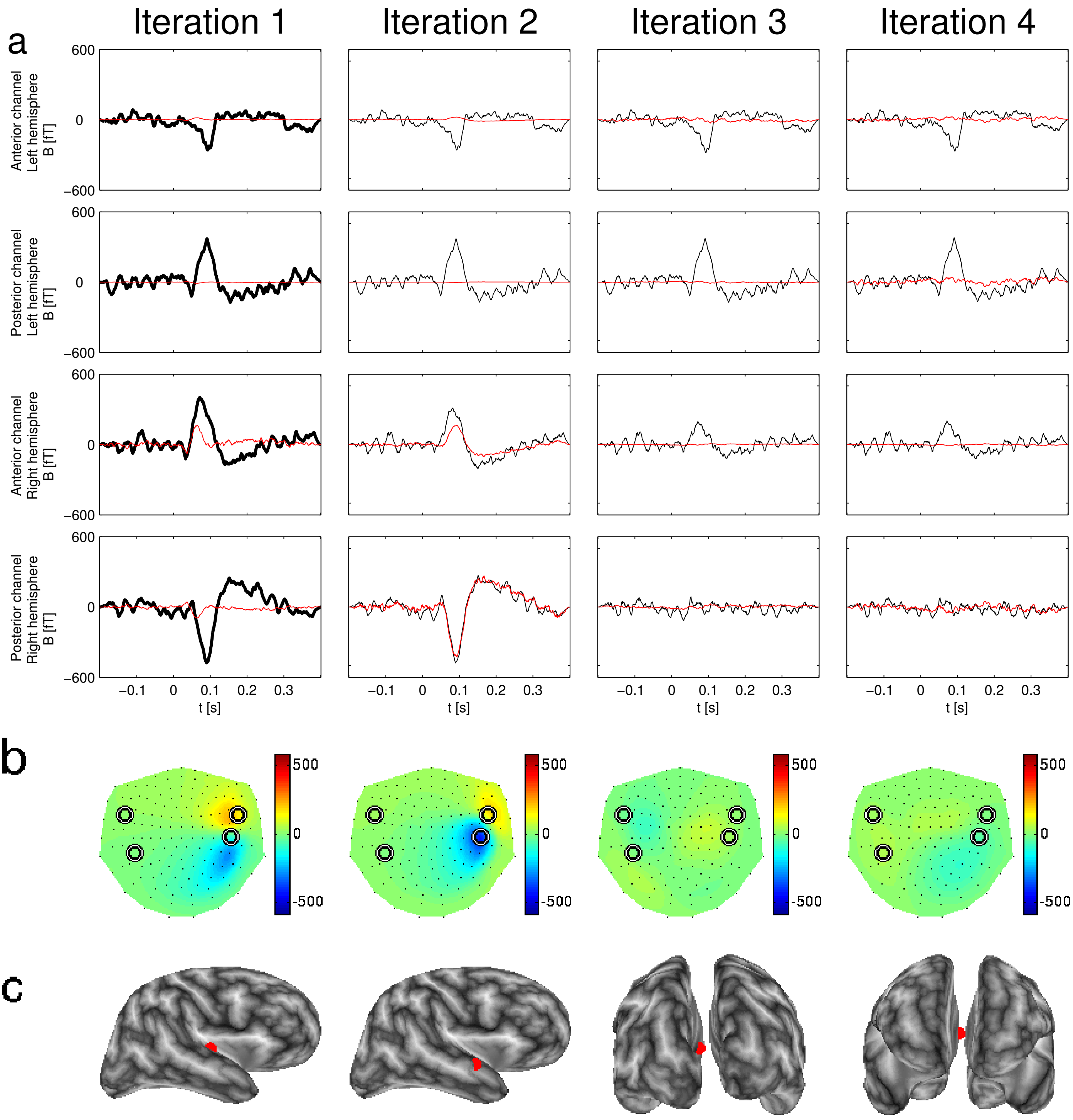

Figure 2: RAP-MUSIC for 48 trials, i.e. every fourth trial from the original dataset of 190 trials.

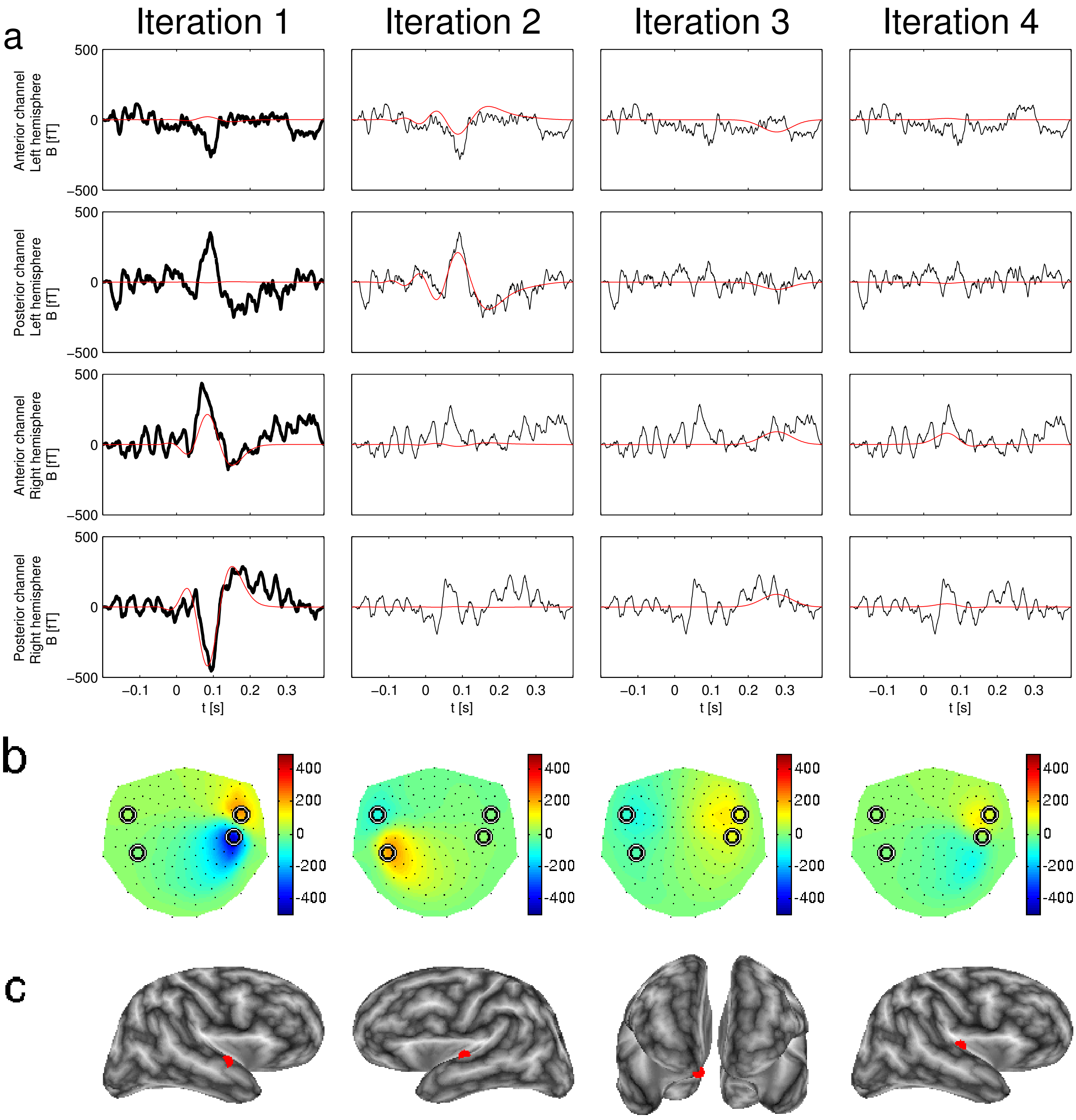

Figure 3: STMP for 24 trials, i.e. every eighth trial from the original dataset of 190 trials.

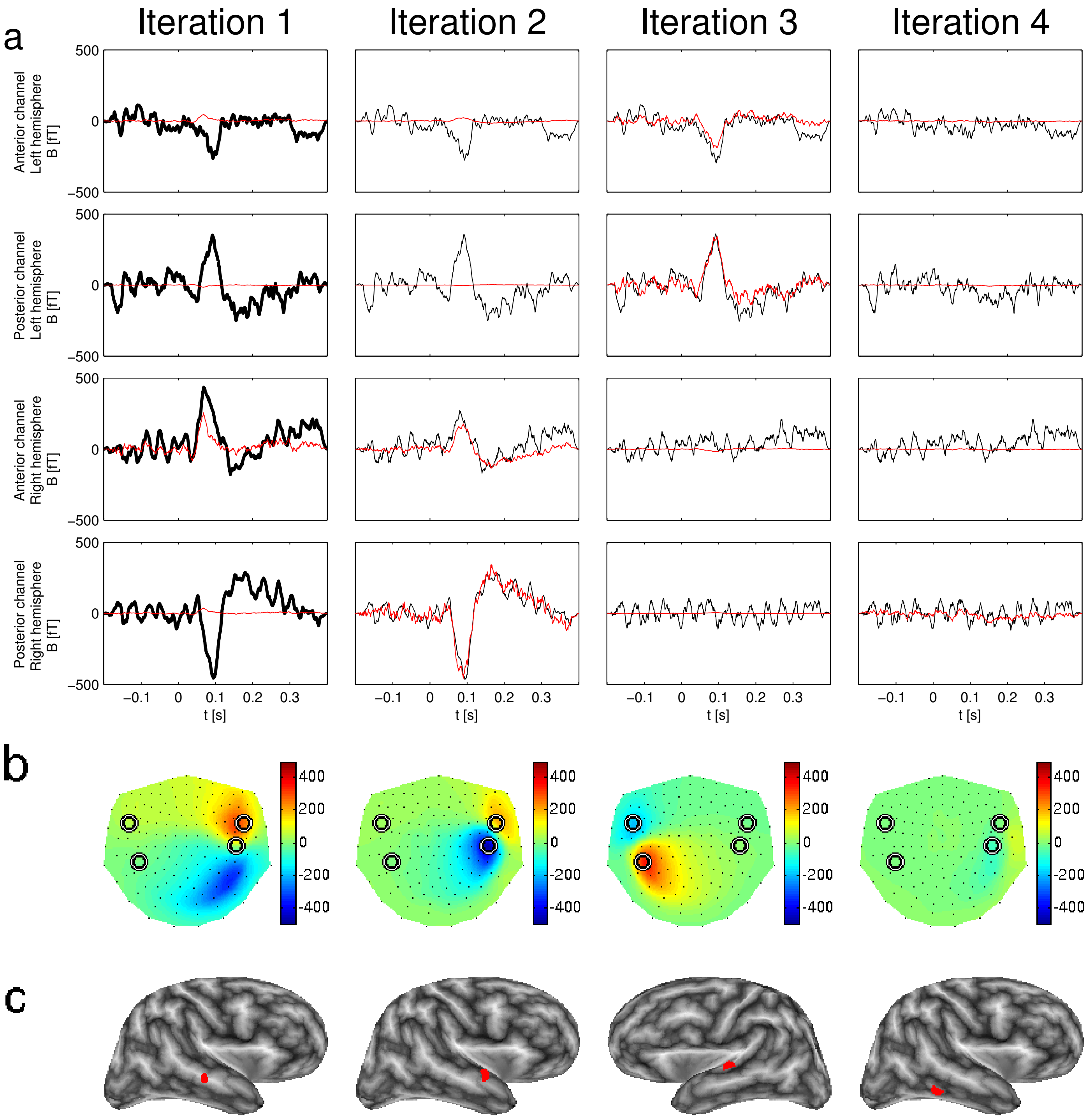

Figure 4: RAP-MUSIC for 24 trials, i.e. every eighth trial from the original dataset of 190 trials.
